# Supplementary material for: Key challenges facing data-driven multicellular systems biology
Source: Gigascience. 2019 Oct 24;8(10):giz127. doi: 10.1093/gigascience/giz127 (PMC6812467; doi:10.1093/gigascience/giz127)
Supplement: giz127_GIGA-D-18-00182_Original_Submission [file giz127_giga-d-18-00182_original_submission.pdf]

GigaScience

Key challenges facing data-driven multicellular systems biology

--Manuscript Draft--

**Manuscript Number:**	GIGA-D-18-00182
**Full Title:**	Key challenges facing data-driven multicellular systems biology
**Article Type:**	Review
**Funding Information:**	Breast Cancer Research Foundation (US) — Dr Paul Macklin<br>Jayne Koskinas Ted Giovanis Foundation for Health and Policy — Dr Paul Macklin<br>National Cancer Institute (1R01CA180149) — Dr Paul Macklin<br>National Cancer Institute (5U54CA143907) — Dr Paul Macklin<br>National Science Foundation (1720625) — Dr Paul Macklin
**Abstract:**	Increasingly sophisticated experiments, coupled with large-scale computational models, have the potential to systematically test biological hypotheses to drive our understanding of multicellular systems. In this short review, we explore key challenges that must be overcome to achieve robust, repeatable data-driven multicellular systems biology. If these challenges can be solved, we can grow beyond the current state of isolated tools and datasets to a community-driven ecosystem of interoperable data, software utilities, and computational modeling platforms. Progress is within our grasp, but it will take community (and financial) commitment.
**Corresponding Author:**	Paul Macklin, Ph.D.<br>Indiana University<br>Bloomington, IN UNITED STATES
**Corresponding Author Secondary Information:**	
**Corresponding Author's Institution:**	Indiana University
**Corresponding Author's Secondary Institution:**	
**First Author:**	Paul Macklin, Ph.D.
**First Author Secondary Information:**	
**Order of Authors:**	Paul Macklin, Ph.D.
**Order of Authors Secondary Information:**	
**Additional Information:**	
**Question**	**Response**
Are you submitting this manuscript to a special series or article collection?	No
**Experimental design and statistics**<br><br>Full details of the experimental design and statistical methods used should be given in the Methods section, as detailed in our Minimum Standards Reporting Checklist. Information essential to interpreting the data presented should be made available in the figure legends.	No

Powered by Editorial Manager® and ProduXion Manager® from Aries Systems Corporation

Have you included all the information requested in your manuscript?	
**Resources**<br><br>A description of all resources used, including antibodies, cell lines, animals and software tools, with enough information to allow them to be uniquely identified, should be included in the Methods section. Authors are strongly encouraged to cite Research Resource Identifiers (RRIDs) for antibodies, model organisms and tools, where possible.<br><br>Have you included the information requested as detailed in our Minimum Standards Reporting Checklist?	No
**Availability of data and materials**<br><br>All datasets and code on which the conclusions of the paper rely must be either included in your submission or deposited in publicly available repositories (where available and ethically appropriate), referencing such data using a unique identifier in the references and in the “Availability of Data and Materials” section of your manuscript.<br><br>Have you have met the above requirement as detailed in our Minimum Standards Reporting Checklist?	No

built PDF Click here to download Manuscript GigaScience_review.pdf

OXFORD (GIGA)^n SCIENCE

GigaScience, 2018, 1–7

doi: xx.xxxx/xxxx
Manuscript in Preparation
Review

REVIEW

Key challenges facing data-driven multicellular systems biology

Paul Macklin[1,*]

[1]Department of Intelligent Systems Engineering, Indiana University, Bloomington, IN USA

[*]macklinp@iu.edu

Abstract

Increasingly sophisticated experiments, coupled with large-scale computational models, have the potential to systematically test biological hypotheses to drive our understanding of multicellular systems. In this short review, we explore key challenges that must be overcome to achieve robust, repeatable data-driven multicellular systems biology. If these challenges can be solved, we can grow beyond the current state of isolated tools and datasets to a community-driven ecosystem of interoperable data, software utilities, and computational modeling platforms. Progress is within our grasp, but it will take community (and financial) commitment.

Key words: multicellular systems biology; data-driven; challenges; multidisciplinary; open source; open data; data standards; big data; simulations; machine learning

Background

In the past decade, we have seen tremendous advances in measuring, annotating, analyzing, understanding, and even manipulating the systems biology of single cells. Not only can we perform single-cell multi-omics measurements in high throughput (e.g., [1, 2, 3]), but we can manipulate single cells (e.g., by CRISPR systems [4]), and we can track single-cell histories through novel techniques like DNA barcoding [5].

As these techniques mature, new questions arise: How do single-cell characteristics affect multicellular systems? How do cells communicate and coordinate? How do systems of mixed cell types create specific spatiotemporal and functional patterns in tissues? How do multicellular organisms cope with single-cell mutations and other errors? Conversely, given a set of functional design goals, how do we manipulate single-cell behaviors to achieve our design objectives? Questions like these are at the heart of ***multicellular systems biology***. As we move from understanding to designing multicellular behavior, we arrive at ***multicellular systems engineering***.

High-throughput multiplex experiments are poised to create incredibly high-resolution datasets describing the molecular and behavioral state of many cells in three-dimensional tissue systems. Computational modeling—including dynamical simulation models and machine learning approaches—can help make sense of these data.

Modelers "translate" a biologist's current set of hypotheses into simulation rules, then simulate the system forward in time. They compare these results to experimental data to evaluate the hypotheses, and refine them until simulations match experiments [6]. Computational models allow us to ask "what if" questions [7]. *What if* we added a new cell type to the mix? *What if* we spliced in a new signaling pathway? How would our system change?

Machine learning and bioinformatics complement the dynamical modeling approach: analyses of large datasets—especially when annotated with expert-selected biological and clinical features—can be mined to discover new relationships between single-cell states and behaviors, multicellular organization, and emergent function. This, in turn, can drive new hypotheses in simulation models. Moreover, machine learning can provide novel analyses of simulation data, increasing what we learn from the efforts.

Examples of these approaches appear largely as isolated efforts. Most groups seek out their own data sources (previously published data and tailored experiments), build their own models, and perform their own analyses. Much of this work uses in-house tools created to work on datasets with *ad*

Compiled on: May 19, 2018.
Draft manuscript prepared by the author.

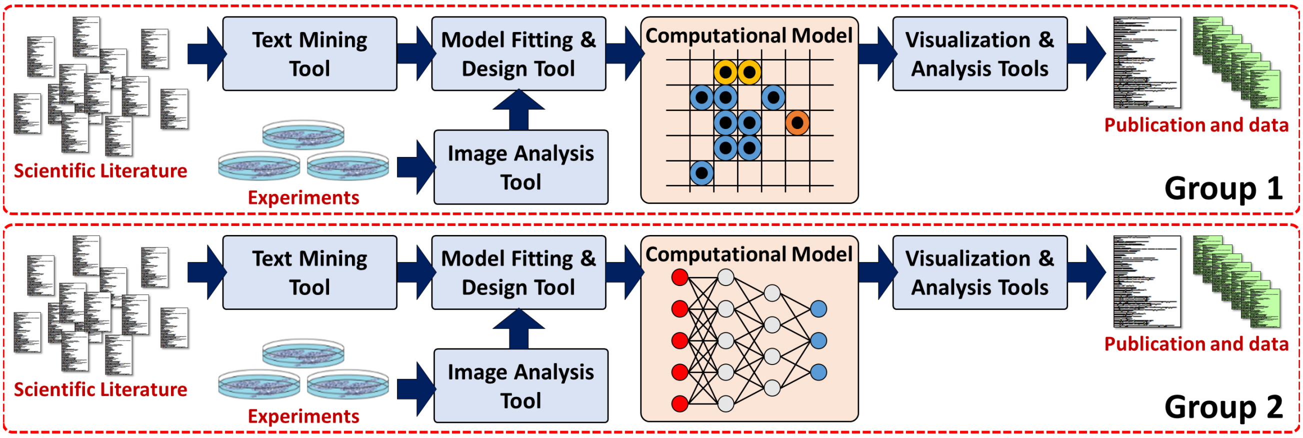

Figure 1. Currently, data-driven workflows are largely parallel, with custom-made, incompatible data and tools.

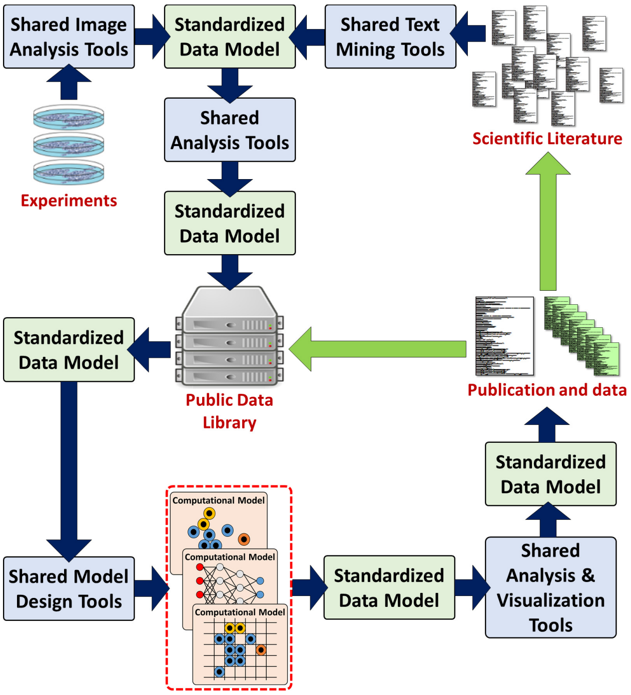

Figure 2. If the community can overcome key challenges, an ecosystem of interoperable computational modeling, analysis, configuration, visualization, and other tools could work on community-curated data and aggregate insights from many sources.

hoc, non-interoperable data elements. See Figure 1. Thus, any one group's work is by and large incompatible with any other group's, hindering or altogether preventing replication studies and modular reuse of valuable data and software tools.

It doesn't have to be this way. If we could solve key challenges, we could move beyond single-lab efforts to a community built around compatible data and software. Multiple experimental labs could pool their efforts to characterize common experimental model systems, and record their data in centralized repositories. With a shared "data language," labs could cooperatively build better simulation, analysis, and visualization tools. Multiple computational labs could build models off of these shared data and tools, find new biological insights, and feed them back into the community. See Figure 2.

In this review, we will explore key challenges that must be overcome before we can create an ecosystem of interoperable data and tools for multicellular systems biology.

Key Challenges

Shared multicellular data standards

Data arising from high-throughput experiments need to be machine readable and stored in interoperable formats with biologically meaningful data elements. We need to move beyond shared drives of raw images and spreadsheets, to extracted biological data elements that are useful for building models and machine learning. We need to store not only averaged cell data, but also single-cell states for many cells at multiple time points. Measurements lose meaning without context: data must be stored with metadata including detailed cell line and (molecular) growth media details, biophysical culture conditions, who performed the measurements, what instruments were used, and what software tools were used for analysis.

Current progress

Great strides have been made towards this challenge. The Open Microscopy Environment (OME) has emerged as a biological image standard with extensive metadata [8], which has helped to make scientific instruments more interoperable. The ISA-Tab format [9] functions as a rich online file system: provenance and other metadata are bundled with raw data of any file type, allowing the contents to be indexed and searched without detailed knowledge of the data formatting. This has facilitated the creation of large databases of very heterogeneous data (such as GigaDB [10]), and it enables simple data exchange due to its support for many data types.

While these formats facilitate file-level interoperability, they do not encode extracted biological data elements. Protocols.IO was developed to share detailed experimental protocols [11], which can be cited in journal publications to help improve repeatability and reproducibility. However, the protocols are human-readable checklists; they do not use a machine-readable controlled vocabulary of growth factors and other culture conditions.

Ontologies such as the Medical Subject Headings (MeSH) [12, 13] and the Cell Behavior Ontology (CBO) can annotate many biological concepts [14], but they serve as controlled vocabularies rather than standardized data formats. The Systems Biology Markup Language (SBML) is a well-established standard for single-cell systems biology [15], and efforts such as SBML-Dynamic are working to extend SBML to multicellular models. Domain experts in computational biology, experimental biology, and data science worked together to draft MultiCellDS, a standard for multicellular data [16]. MultiCellDS has a highly extensible representation of single-cell phenotype built from a variety of ontologies such as MeSH and CBO, which can

be used to represent highly multiplex data (e.g., [1]) for many cells, along with metadata and microenvironmental context.

Future

None of these efforts has completely addressed this challenge. Ultimately, we should combine and extend them into a unified data format. ISA-Tab could bundle image data (using OME) and extracted biological features (e.g., with MultiCellDS), while storing experimental protocol details with a controlled vocabulary growing out of Protocols.IO.

Shared multicellular observational representations

Beyond quantitative measurements like cell division rates, we need a machine-readable encoding of qualitative observations and insights derived from raw biological data: when cells are in condition *X*, they do *Y*. When cells of type *X* and *Y* interact by contact, they tend to do *Z*. When cell line *X* looks like *Y* in an experiment, the cell culture medium lacked factor *Z*.

Labs and clinics are replete with such examples of hard-won knowledge, but until we can systematically record them, these insights will remain siloed, isolated, and destined to be relearned, lab by lab. If we could consistently record qualitative observations, we could progress from single-cell measurements to multicellular systems understanding, including annotation of critical cell-cell interactions.

Until we can specify “correct” model behavior with machine-readable annotations, our simulation studies will be rate-limited to how quickly humans can view simulations and assess them as more or less “realistic.” How do we say, in a generalized way, that a simulated tumor stays compact or becomes invasive? How do we know if a simulated developmental process has the “right” amount of branching? What does it mean for simulated image *X* to “look like” experimental image *Y*, given that both the simulation and the experiment are single instances of stochastic processes? If we cannot record the qualitative behavior of simulations and experiments, we cannot automate processes to compare them.

Current progress

Progress on this challenge has been limited. The CBO [14] has developed a good starting vocabulary for observed cell behaviors. Extensions of SBML [15] could also potentially represent some of these multicellular and multiscale observations. Tailored image processing has been applied to individual investigations to extract (generally quantitative) representations, although to date we have seen few (if any) qualitative descriptors generated by systematic image analysis.

Future

This area seems ripe for machine learning: given a set of qualitative descriptors like “compact” versus “invasive,” “mixed” versus “separated,” “growing” versus “shrinking” or “steady,” a neural network could be trained to human classifications of experimental and simulation data. High-throughput multicellular simulators (e.g., [6]) could create large sets of training data in standardized formats with clear ground truths. Machine vision could also be be used to analyze time series of multicellular data. These annotations could give rise to metrics that help us systematically compare the behavior of one simulation with another, or to determine which simulation (in a set of hundreds or thousands of simulations) behaves most like an experiment.

Standards support in computational tools

For data standards to be truly useful, they must be broadly supported by a variety of interoperable tools.

Current progress

Single-cell systems biology has already shown the enabling role of stable data standards [17]: once SBML crystallized as a stable data language, a rich and growing ecosystem of data-compatible simulation and analysis software emerged. Multicellular systems biology has not yet reached this point: most computational models have custom configuration and output formats, sometimes with customized extensions of SBML to represent single-cell systems biology [16].

Future

If a multicellular data standard emerges, key open source projects [17] can implement read and write support in their software, either “natively” (i.e., at run-time), or as data converters. Hackathons or similar hosted workshops could facilitate this work. Ontologists need to provide user-friendly data bindings to simplify these development efforts. If standards are to be supported more broadly than just major open source packages, we must remember that most scientific software is created with little formal software engineering training; the data bindings must be well-documented, have simple syntax, and require minimal installation effort.

Shared tools to configure models and explore data

It is not enough to simply read and write data into individual tools. We must reverse the current “lock in” effect: because multicellular modeling software is difficult to learn, users (and often entire labs) focus their training on a single modeling approach. Because of this, replication studies are rare, even when a study’s source code and data are openly available.

To solve this, we need user-friendly tools to import and set biological and biophysical parameters, design the virtual geometry, and write standardized configuration files that initialize many modeling frameworks. Users could run models in multiple software packages, replicate the work of others, and avoid software-specific artifacts that can bias their conclusions.

Shared software to read, analyze, compare, and visualize outputs from multiple modeling packages could reduce the learning curve for new software. If the shared data exploration and analysis tools were written to work on a common format that includes segmented experimental data, they could also be used to explore experimental data, make and annotate new observations, and motivate new model hypotheses.

Current progress

Without a common format for multicellular simulation data, there has been little opportunity to develop shared tools for configuring, running, and visualizing multicellular simulations. Some individual simulation packages such as Morpheus [18] and CompuCell3D [19] have user-friendly graphical model editors, but they are currently limited to their individual user communities and not compatible with other simulation packages [17]. Commercially-backed open source software such as Kitware’s ParaView [20] is commonly used to visualize multicellular simulation data, but only by writing customized, simulation-tailored data importers. ParaView is generally not used to visualize biological data.

Future

It will be difficult to make progress on this challenge without stable standards for multicellular input and output data. How-

ever, progress could be made using current draft standards, such as MultiCellDS [16]. ParaView could use customized plugins to support emerging standards for multicellular data. If projects like Morpheus implemented standards, their graphical model editors could become valuable community resources.

Hackathons can help to rapidly prototype new tools (particularly if they are paired with benchmark datasets), but they must aim to create well-documented, engineered software that are maintained in the long term. We may need new funding paradigms to support small open source teams.

High-quality, multiscale benchmarking datasets

Once we have standardized data formats and an ecosystem of compatible software to support them, we need high-quality datasets to drive the development of computational models. The ideal datasets would sufficiently resolve single-cell morphologies and multi-omic states in 3-D tissues, along with microenvironmental context (e.g., spatial distribution of oxygen).

To capture the behavioral states of cells, we need standard immunohistochemical panels that capture multiple dimensions of cell phenotype: cycle status, metabolism, death, motility (including markers for the leading edge), adhesiveness, cell mechanics, polarization, and more. We will need to capture these details simultaneously in many cells at multiple time points, using massively multiplexed technologies.

These datasets would be used to formulate model hypotheses and assumptions (through data exploration using standardized tools), to train models, and to evaluate them. Moreover, as the community develops new computational models, they could be evaluated against benchmark datasets. Benchmark datasets are domain-specific: separate datasets are needed for developmental biology, avascular and vascular tumor growth, autoimmune diseases, and other problems. It is important that these datasets are easily accessible with open data licenses to promote the broadest use possible. Adhering to FAIR (Findability, Accessibility, Interoperability, and Reusability) data principles would be ideal [21].

Current progress

Cancer biology has made perhaps the greatest progress on this challenge, where the NIH-funded Cancer Genome Atlas hosts many genomic, microscopy, and other large datasets [22]. Typically, these consist of many samples at a single time, rather than time course data. Highly multiplex multicellular data are generally not available. DREAM challenges have assembled high-quality datasets to drive model development (through competitions) [23], but these have not typically satisfied the multiplex, time series ideals outlined above. Private foundations are using cutting-edge microscopy to create high-quality online datasets (e.g., the Allen Cell Explorer Project [24]).

The technology for highly-multiplexed measurements is steadily improving: CyTOF-based immunohistochemistry (e.g., as in Levenson et al. [1]) can stain for panels of 30-50 immunomarkers on single slides at 1-2 μm resolution or better. There are no standardized panels to capture the gamut of phenotypic behaviors outlined above. Social media discussions (e.g., [25]) have helped to drive community dialog on difficult phenotypic parameters, but no clear consensus has emerged for a “gold standard” panel of immunostains.

Future

Workshops of leading biologists should assemble the “dream panel” of molecular markers. Consortia of technologists will need to reliably implement these multi-parameter panels in experimental workflows [1]. Workshops of bioinformaticians, data scientists, and modelers will be needed to “transform” these raw data into standardized datasets for use in models. All this will require federal or philanthropic funding, and contributions by multiple labs. Social media has great potential for public brainstorming, disseminating resources, and recruiting new contributors. Hackathons could help drive the “translation” of raw image data into standardized datasets, while developing tools that automate the process.

Community-curated public data libraries

We need “public data libraries” to store and share high-quality, standardized data. Data should not be static: the community should continually update data to reflect scientific advances, with community curation to ensure data quality. Public libraries must not only store raw image data and extracted biological parameters, but also qualitative observations and human insights. The public libraries should host data at multiple stages of publication: preliminary data (which may or may not be permanently archived), datasets under construction (i.e., the experiments are ongoing), data associated with a preprint or a paper in review, and data associated with a published work. Public data libraries should encourage versioned post-publication refinement. Lastly, public data libraries need to be truly public by using licenses (e.g., Creative Commons CC0 or CC-BY) that encourage new derivative works, as well as aggregation into larger datasets.

Current progress

Numerous data portals exist, and more are emerging. Many are purpose-built for specific communities, such as the Cancer Genome Atlas [22]. Others like GigaDB [10] and DRYAD [26] allow users to post self-standing datasets with unique DOIs to facilitate data reuse and attribution. These repositories are free for access, thus increasing the reach and impact of hosted data, but the data contributors must pay at the time of data publication. The fees often include editorial and technical assistance while ensuring long-term data availability.

Even within single data hosting repositories, individual datasets are largely disconnected and mutually non-interoperable beyond ISA-Tab compatibility. Thus, individual hosted datasets and studies are generally not bridged and recombined. Moreover, the datasets are usually static after publication, rather than actively curated and updated. BioNumbers has long served as a searchable resource of user-contributed biological parameters [27], but it lacks a unified data model. The MultiCellDS project proposed *digital cell lines*, which aggregate measurements from many sources for a single cell type [16]. Digital cell lines were intended to be continually updated and curated by the community, so that low-quality measurements could be replaced by better measurements as technology advances. However, this effort is currently manual, with no single, easily searchable repository for its pilot data.

An unfortunate consequence of the current data hosting model is that all the burden rests on data donors: they generate the data, format it to standards, assemble it, document it, upload it, and then pay the hosting and scientific publication costs. This is a classic case of the tragedy of the commons: it is easy to benefit from shared resources, but costly to contribute. Most repositories have fee waivers for scientists in low-income nations, but small and underfunded labs and citizen scientists are still at a disadvantage.

Future

We need to develop more unified, financially stable and scalable repositories that can bridge fields and collect our knowledge. The repositories need to be community curated and continually improved, rather than static. They need to place less effort and

financial burden on those who are donating data.

Solutions to this challenge may well originate outside the bioinformatics community. Library scientists have longstanding domain expertise in collecting and curating knowledge across disciplines in unified physical libraries: this expertise would undoubtedly benefit any efforts to create public data libraries. The tremendous success of Wikipedia [28] in hosting its own image and video resources on Wikimedia Commons [29]—at no cost to contributors—could be a very good model. bioRxiv [30] has been similarly successful in hosting preprints at no cost to authors. Both of these have relied upon a combination of public donations, federal support, and philanthropy, channeled through appropriate nonprofit structures.

Lastly, to ensure robustness and sustainability, we need to encourage data mirroring with global searchability, and promote a culture that values and properly cites all contributions to shared knowledge: data generation, data analysis, and data curation. While badges can help [31, 32], we must ensure that data users can easily cite all these contributions in papers, that impact metrics reflect the breadth of contributions, and that tenure and other career processes truly value all contributions to community knowledge resources.

Quality and curation standards

Community-curated public libraries face new questions: how can we consistently decide which data are worth saving? How do we determine if a new measurement is better than an old one? How do we monitor quality? Can we automatically trust one lab's data contributions based upon prior contributions? And who gets to make these decisions?

Current progress

Little to none, aside from uncertainty quantification.

Future

This challenge is as much cultural as it is technical. We will need to hold workshops of leading biologists to identify community values and standards for assessing different measurement types. The community will need to determine if "gold standards" can be devised for comparing measurements.

Linking data to models

We need to connect data to computational models. Data modelers should help design experiments, to determine what variables are needed to build useful models. We need to determine how to "map" biological measurements to model parameters.

Current progress

This challenge is currently being addressed on a study-by-study basis. Individual teams design experiments, devise their own model calibration methods, formulate model evaluation metrics, and create their own tools to analyze and compare experimental and simulation data.

Future

This challenge is both technical and cultural. Mathematicians, biologists, data scientists, and others will need to work together to determine what it means for an inherently stochastic simulation model to match to match an experiment. Any progress in creating standardized data elements and annotating multicellular systems behaviors will surely help in creating metrics to compare experimental and computational models. Once standardized biological parameters are extracted to create benchmark datasets, machine learning could help drive more systematic mappings from extracted biological parameters to computational model inputs.

Conclusions

The time is ripe for data-driven multicellular systems biology and engineering. Technological advances are making it possible to create high-resolution, highly multiplex multicellular datasets. Computational modeling platforms—including simulation and machine learning approaches—have advanced considerably, and they are increasingly available as open source [17, 33]. Supercomputing resources are amplifying the power of these computational models, while cloud resources are making them accessible to all [34].

If we can solve these key challenges, we will connect big multicellular datasets with computational technologies to accelerate our understanding of biological systems.

Some of the challenges are largely technical, such as creating data standards. Others are more cultural, such as shaping community values for data curation. All of the challenges share a need for community investment: developing and sharing compatible tools and data, hosting data, curating public data libraries, and ultimately funding these worthwhile efforts. Many groups are already contributing pieces of this puzzle, often with little financial support. In the future, we must reduce the individual burden in creating community goods. We may need newer, more rapid funding paradigms to help support and harden new software tools, scaling from small but simple proposals to the current large software grant mechanisms (which tend to have low funding rates). We may need to fund software labs rather than software projects, to encourage rapid response to emerging community needs.

We are on the cusp of accelerated, data-driven biological discovery of how cells work together, how they build things, and how this breaks to cause disease. If you are working towards solving any of these challenges (or if you have new ones to pose!), please consider sharing your advances here.

Declarations

List of abbreviations

**CBO**	Cell Behavior Ontology
**CC0**	Creative Commons public domain license
**CC-BY**	Creative Commons attribution license
**CyTOF**	Cytometry by Time Of Flight
**DOI**	Digital Object Identifier
**DREAM**	Dialogue for Reverse Engineering Assessments and Methods
**FAIR**	Findability, Accessibility, Interoperability, and Reusability
**ISA-Tab**	Investigation-Study-Assay tabular format
**MeSH**	Medical Subject Headings
**NIH**	(U.S.) National Institutes of Health
**OME**	Open Microscopy Environment
**SBML**	Systems Biology Markup Language

Competing Interests

The author declares that he has no competing interests.

Funding

PM's work to develop computational tools and data standards for multicellular systems biology was funded by the Breast Can-

cer Research Foundation (PIs Agus, Ewald, Gilkes and Macklin) and the Jayne Koskinas Ted Giovanis Foundation for Health and Policy (PIs Ewald and Gilkes), the National Science Foundation (PI Fox, 1720625), and the National Cancer Institute (PIs Agus, Atala, and Soker, 1R01CA180149; PI Hillis, 5U54CA143907).

Author's Contributions

PM conceptualized and wrote the manuscript.

Acknowledgements

PM thanks Nicole Nogoy and the staff of *GigaScience* for the opportunity to write this review, and for editorial support.

Author's information

PM has worked for over ten years in computational multicellular systems biology, with a focus on cancer biology and tissue engineering. He has written several open source tools for the field, including BioFVM (a multi-substrate diffusion solver for biochemical cell-cell communication) [35], PhysiCell (a 3-D agent-based modeling toolkit) [36], and MultiCellDS (a draft multicellular data standard) [16]. He is an Associate Professor of Intelligent Systems Engineering at Indiana University.

References

1. Levenson RM, Borowsky AD, Angelo M. Immunohistochemistry and mass spectrometry for highly multiplexed cellular molecular imaging. Laboratory Investigation 2015;95:397. https://dx.doi.org/10.1038/labinvest.2015.2.
2. Kim M, Rai N, Zorraquino V, Tagkopoulos I. Multi-omics integration accurately predicts cellular state in unexplored conditions for Escherichia coli. Nat Comm 2016;7:13090. http://dx.doi.org/10.1038/ncomms13090.
3. Norris JL, Farrow MA, Gutierrez DB, Palmer LD, Muszynski N, Sherrod SD, et al. Integrated, High-Throughput, Multiomics Platform Enables Data-Driven Construction of Cellular Responses and Reveals Global Drug Mechanisms of Action. J Proteome Res 2017;16(3):1364–1375. https://doi.org/10.1021/acs.jproteome.6b01004.
4. Adli M. The CRISPR tool kit for genome editing and beyond 2018;.
5. Blundell JR, Levy SF. Beyond genome sequencing: Lineage tracking with barcodes to study the dynamics of evolution, infection, and cancer. Genomics 2014;104(6, Part A):417 – 430. http://www.sciencedirect.com/science/article/pii/S0888754314001827, experimental evolution and the use of genomics.
6. Ozik J, Collier N, Wozniak J, Macal C, Cockrell C, Friedman S, et al. High-throughput cancer hypothesis testing with an integrated PhysiCell-EMEWS workflow. BMC Bioinformatics 2018 (accepted, see bioRxiv preprint 196709);http://dx.doi.org/10.1101/196709.
7. Macklin P. When Seeing Isn't Believing: How Math Can Guide Our Interpretation of Measurements and Experiments. Cell Sys 2017;5(2):92–4. http://dx.doi.org/10.1016/j.cels.2017.08.005.
8. Goldberg IG, Allan C, Burel JM, Creager D, Falconi A, Hochheiser H, et al. The Open Microscopy Environment (OME) Data Model and XML file: open tools for informatics and quantitative analysis in biological imaging. Genome Biology 2005 May;6(5):R47. https://doi.org/10.1186/gb-2005-6-5-r47.
9. Rocca-Serra P, Brandizi M, Maguire E, Sklyar N, Taylor C, Begley K, et al. ISA software suite: supporting standards-compliant experimental annotation and enabling curation at the community level. Bioinformatics 2010;26(18):2354–2356. http://dx.doi.org/10.1093/bioinformatics/btq415.
10. GigaDB Website; visited 2018-05-17. http://gigadb.org.
11. Teytelman L, Stoliartchouk A, Kindler L, Hurwitz BL. Protocols.io: Virtual Communities for Protocol Development and Discussion. PLOS Biology 2016 08;14(8):1–6. https://doi.org/10.1371/journal.pbio.1002538.
12. Rogers FB. Communications to the Editor. Bull Med Lib Assoc 1963;51(1):114—-6. https://www.ncbi.nlm.nih.gov/pmc/articles/PMC197951/.
13. Lipscomb CE. Medical Subject Headings (MeSH). Bull Med Lib Assoc 2000;88(3):265—-6. https://www.ncbi.nlm.nih.gov/pmc/articles/PMC35238/.
14. Sluka JP, Shirinifard A, Swat M, Cosmanescu A, Heiland RW, Glazier JA. The cell behavior ontology: describing the intrinsic biological behaviors of real and model cells seen as active agents. Bioinformatics 2014;30(16):2367–2374. http://dx.doi.org/10.1093/bioinformatics/btu210.
15. Hucka M, Finney A, Sauro HM, Bolouri H, Doyle JC, Kitano H, et al. The systems biology markup language (SBML): a medium for representation and exchange of biochemical network models. Bioinformatics 2003;19(4):524–531. http://dx.doi.org/10.1093/bioinformatics/btg015.
16. Friedman SH, Anderson ARA, Bortz DM, Fletcher AG, Frieboes HB, Ghaffarizadeh A, et al. MultiCellDS: a community-developed standard for curating microenvironment-dependent multicellular data. bioRxiv 2016;090456. http://dx.doi.org/10.1101/090456, (**Preprint**, preparing for resubmission).
17. Macklin P, Friedman SH, MultiCellDS. Open source tools and standardized data in cancer systems biology. bioRxiv 2018;244319. https://dx.doi.org/10.1101/244319, (part of the NCI CSBC/PS-ON Handbook of Mathematical Oncology).
18. Starruß J, de Back W, Brusch L, Deutsch A. Morpheus: a user-friendly modeling environment for multiscale and multicellular systems biology. Bioinformatics 2014;30(9):1331–1332. http://dx.doi.org/10.1093/bioinformatics/btt772.
19. Swat MH, Thomas GL, Belmonte JM, Shirinifard A, Hmeljak D, Glazier JA. Chapter 13 - Multi-Scale Modeling of Tissues Using CompuCell3D. In: Asthagiri AR, Arkin AP, editors. Computational Methods in Cell Biology, vol. 110 of Methods in Cell Biology Academic Press; 2012.p. 325 – 366. http://dx.doi.org/10.1016/B978-0-12-388403-9.00013-8.
20. Kitware, ParaView Website; visited 2018-05-17. https://www.paraview.org/.
21. Wilkinson MD, Dumontier M, Aalbersberg IJ, Appleton G, Axton M, Baak A, et al. The FAIR Guiding Principles for scientific data management and stewardship. Scientific Data 2016;3:160018. http://dx.doi.org/10.1038/sdata.2016.18.
22. The Cancer Genome Atlas; visited 2018-05-17. https://cancergenome.nih.gov/.
23. DREAM Challenges Project Website; visited 2018-05-17. http://dreamchallenges.org/.
24. Allen Institute, Allen Cell Explorer Project; visited 2018-05-17. http://www.allencell.org/cell-catalog.html.
25. @MAG2ART, @MathCancer, @Alexis_Lomakin, @KidneyMyosin, Twitter discussion on cell motility immunostains; visited 2017-12-13. https://twitter.com/MAG2ART/status/940942422074384384.
26. DRYAD Digital Repository; visited 2018-05-17. https://datadryad.org/.

27. Milo R, Jorgensen P, Moran U, Weber G, Springer M. BioNumbers—the database of key numbers in molecular and cell biology. Nucleic Acids Research 2010;38(Suppl. 1):D750–D753. http://dx.doi.org/10.1093/nar/gkp889.
28. Wikipedia; visited 2018-05-17. https://wikipedia.org.
29. Wikimedia Commons; visited 2018-05-17. https://www.wikimedia.org/.
30. bioRxiv: The Preprint Server for Biology; visited 2018-05-17. https://biorxiv.org.
31. Kidwell MC, Lazarević LB, Baranski E, Hardwicke TE, Piechowski S, Falkenberg LS, et al. Badges to Acknowledge Open Practices: A Simple, Low-Cost, Effective Method for Increasing Transparency. PLOS Biology 2016 05;14(5):1–15. https://doi.org/10.1371/journal.pbio.1002456.
32. Rowhani-Farid A, Allen M, Barnett AG. What incentives increase data sharing in health and medical research? A systematic review. Research Integrity and Peer Review 2017 May;2(1):4. https://doi.org/10.1186/s41073-017-0028-9.
33. Macklin P, Frieboes HB, Sparks JL, Ghaffarizadeh A, Friedman SH, Juarez EF, et al. Progress Towards Computational 3-D Multicellular Systems Biology. In: Rejniak KA, editor. Systems Biology of Tumor Microenvironment, Advances in Experimental Medicine and Biology, vol. 936 Bern, Switzerland: Springer; 2016 (in press).http://dx.doi.org/10.1007/978-3-319-42023-3_12, (invited author: P. Macklin).
34. Madhavan K, Zentner L, Farnsworth V, Shivarajapura S, Zentner M, Denny N, et al. nanoHUB.org: cloud-based services for nanoscale modeling, simulation, and education. Nanotech Rev 2013;2(1):107–17. https://doi.org/10.1515/ntrev-2012-0043.
35. Ghaffarizadeh A, Friedman SH, Macklin P. BioFVM: an efficient, parallelized diffusive transport solver for 3-D biological simulations. Bioinformatics 2016;32(8):1256–8. http://dx.doi.org/10.1093/bioinformatics/btv730.
36. Ghaffarizadeh A, Heiland R, Friedman SH, Mumenthaler SM, Macklin P. PhysiCell: an open source physics-based cell simulator for 3-D multicellular systems. PLoS Comput Biol 2018;14(2):e1005991. http://dx.doi.org/10.1371/journal.pcbi.1005991.
