# Supplementary material for: Key challenges facing data-driven multicellular systems biology
Source: Gigascience. 2019 Oct 24;8(10):giz127. doi: 10.1093/gigascience/giz127 (PMC6812467; doi:10.1093/gigascience/giz127)
Supplement: giz127_GIGA-D-18-00182_Revision_1 [file giz127_giga-d-18-00182_revision_1.pdf]

GigaScience

Key challenges facing data-driven multicellular systems biology

--Manuscript Draft--

**Manuscript Number:**	GIGA-D-18-00182R1
**Full Title:**	Key challenges facing data-driven multicellular systems biology
**Article Type:**	Review
**Funding Information:**	Breast Cancer Research Foundation (US) — Dr Paul Macklin<br>Jayne Koskinas Ted Giovanis Foundation for Health and Policy — Dr Paul Macklin<br>National Cancer Institute (1R01CA180149) — Dr Paul Macklin<br>National Cancer Institute (5U54CA143907) — Dr Paul Macklin<br>National Science Foundation (1720625) — Dr Paul Macklin<br>National Cancer Institute (U01-CA232137-01) — Dr Paul Macklin
**Abstract:**	Increasingly sophisticated experiments, coupled with large-scale computational models, have the potential to systematically test biological hypotheses to drive our understanding of multicellular systems. In this short review, we explore key challenges that must be overcome to achieve robust, repeatable data-driven multicellular systems biology. If these challenges can be solved, we can grow beyond the current state of isolated tools and datasets to a community-driven ecosystem of interoperable data, software utilities, and computational modeling platforms. Progress is within our grasp, but it will take community (and financial) commitment.
**Corresponding Author:**	Paul Macklin, Ph.D.<br>Indiana University<br>Bloomington, IN UNITED STATES
**Corresponding Author Secondary Information:**	
**Corresponding Author's Institution:**	Indiana University
**Corresponding Author's Secondary Institution:**	
**First Author:**	Paul Macklin, Ph.D.
**First Author Secondary Information:**	
**Order of Authors:**	Paul Macklin, Ph.D.
**Order of Authors Secondary Information:**	
**Response to Reviewers:**	Please see attached letter.
**Additional Information:**	
**Question**	**Response**
Are you submitting this manuscript to a special series or article collection?	No
**Experimental design and statistics**<br><br>Full details of the experimental design and statistical methods used should be given in the Methods section, as detailed in our	No

Minimum Standards Reporting Checklist. Information essential to interpreting the data presented should be made available in the figure legends.<br><br>Have you included all the information requested in your manuscript?	
**Resources**<br><br>A description of all resources used, including antibodies, cell lines, animals and software tools, with enough information to allow them to be uniquely identified, should be included in the Methods section. Authors are strongly encouraged to cite Research Resource Identifiers (RRIDs) for antibodies, model organisms and tools, where possible.<br><br>Have you included the information requested as detailed in our Minimum Standards Reporting Checklist?	No
**Availability of data and materials**<br><br>All datasets and code on which the conclusions of the paper rely must be either included in your submission or deposited in publicly available repositories (where available and ethically appropriate), referencing such data using a unique identifier in the references and in the “Availability of Data and Materials” section of your manuscript.<br><br>Have you have met the above requirement as detailed in our Minimum Standards Reporting Checklist?	No

revised manuscript
Click here to access/download;Manuscript;main.pdf
Click here to view linked References

GigaScience, 2019, 1–8

doi: xx.xxxx/xxxx
Manuscript in Preparation
Review

REVIEW

Key challenges facing data-driven multicellular systems biology

Paul Macklin[1,*]

[1]Department of Intelligent Systems Engineering, Indiana University, Bloomington, IN USA

[*]macklinp@iu.edu

Abstract

Increasingly sophisticated experiments, coupled with large-scale computational models, have the potential to systematically test biological hypotheses to drive our understanding of multicellular systems. In this short review, we explore key challenges that must be overcome to achieve robust, repeatable data-driven multicellular systems biology. If these challenges can be solved, we can grow beyond the current state of isolated tools and datasets to a community-driven ecosystem of interoperable data, software utilities, and computational modeling platforms. Progress is within our grasp, but it will take community (and financial) commitment.

Key words: multicellular systems biology; data-driven; challenges; multidisciplinary; open source; open data; data standards; big data; simulations; machine learning

Background

In the past decade, we have seen tremendous advances in measuring, annotating, analyzing, understanding, and even manipulating the systems biology of single cells. Not only can we perform single-cell multi-omics measurements in high throughput (e.g., [1, 2, 3]), but we can manipulate single cells (e.g., by CRISPR systems [4]), and we can track single-cell histories through novel techniques like DNA barcoding [5].

As these techniques mature, new questions arise: How do single-cell characteristics affect multicellular systems? How do cells communicate and coordinate? How do systems of mixed cell types create specific spatiotemporal and functional patterns in tissues? How do multicellular organisms cope with single-cell mutations and other errors? Conversely, given a set of functional design goals, how do we manipulate single-cell behaviors to achieve our design objectives? Questions like these are at the heart of ***multicellular systems biology***. As we move from understanding to designing multicellular behavior, we arrive at ***multicellular systems engineering***.

High-throughput multiplex experiments are poised to create incredibly high-resolution datasets describing the molecular and behavioral state of many cells in three-dimensional tissue systems. Computational modeling—including dynamical simulation models and machine learning approaches—can help make sense of these data.

Modelers "translate" a biologist's current set of hypotheses into simulation rules, then simulate the system forward in time. They compare these results to experimental data to evaluate the hypotheses, and refine them until simulations match experiments [6, 7]. Computational models allow us to ask "what if" questions [8]. *What if* we added a new cell type to the mix? *What if* we spliced in a new signaling pathway? How would our system change?

Machine learning and bioinformatics complement the dynamical modeling approach: analyses of large datasets—especially when annotated with expert-selected biological and clinical features—can be mined to discover new relationships between single-cell states and behaviors, multicellular organization, and emergent function. This, in turn, can drive new hypotheses in simulation models. Moreover, machine learning can provide novel analyses of simulation data, increasing what we learn from the efforts.

Examples of these approaches appear largely as isolated efforts. Most groups seek out their own data sources (previously published data and tailored experiments), build their own models, and perform their own analyses. Much of this work uses in-house tools created to work on datasets with *ad*

Compiled on: September 27, 2019.
Draft manuscript prepared by the author.

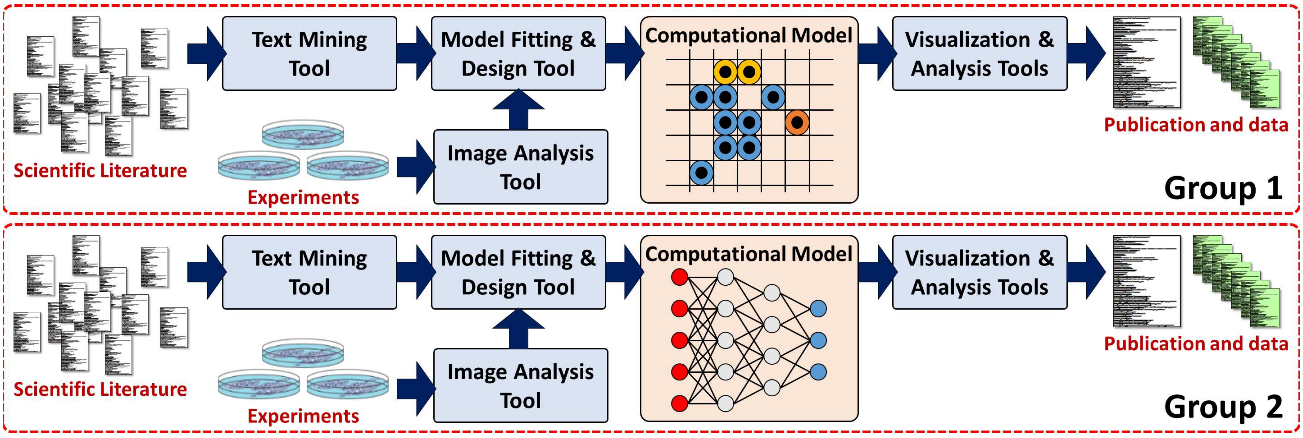

Figure 1. Currently, data-driven workflows are largely parallel, with custom-made, incompatible data and tools.

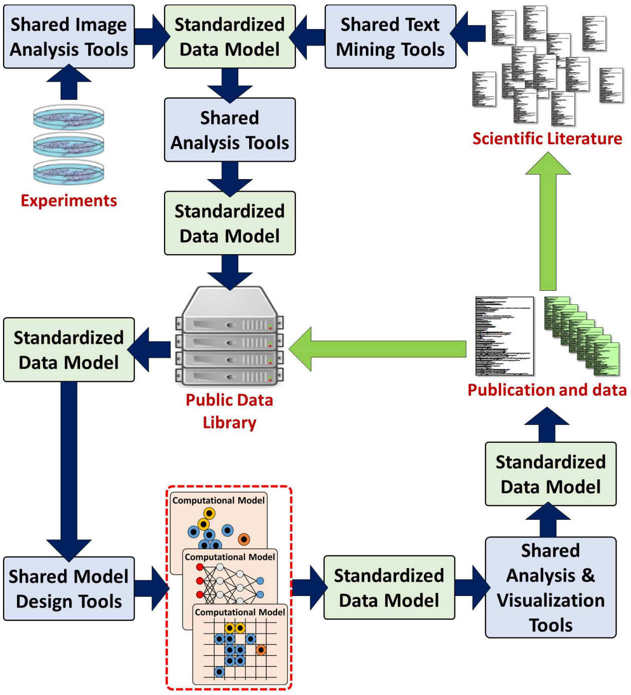

Figure 2. If the community can overcome key challenges, an ecosystem of interoperable computational modeling, analysis, configuration, visualization, and other tools could work on community-curated data and aggregate insights from many sources.

hoc, non-interoperable data elements. See Figure 1. Thus, any one group's work is by and large incompatible with any other group's, hindering or altogether preventing replication studies and modular reuse of valuable data and software.~~tools.~~

It doesn't have to be this way. If we could solve key challenges, we could move beyond single-lab efforts to a community built around compatible data and software. Multiple experimental labs could pool their efforts to characterize common experimental model systems, and record their data in centralized repositories. With a shared "data language," labs could cooperatively build better simulation, analysis, and visualization tools. Multiple computational labs could build models off of these shared data and tools, find new biological insights, and feed them back into the community. See Figure 2.

In this review, we will explore some key challenges that we need to ~~must be~~ overcome before we can reach the full potential of ~~create~~ an ecosystem of interoperable data and tools for multicellular systems biology.

While the challenges are not presented in any ranked order of importance or priority, they progress from the concrete challenges of standardized data representation and knowledge capture to community resources we could build with standardized data. We do not need to address these challenges sequentially. One of the great strengths of open research communities is that progress can occur by many groups in parallel, each contributing according to their individual skills, resources, and interests.

Key Challenges

1. Shared multicellular data standards

Data arising from high-throughput experiments need to be machine readable and stored in interoperable formats with biologically meaningful data elements. We need to move beyond shared drives of raw images and spreadsheets, to extracted biological data elements that are useful for building models and machine learning. We need to store not only averaged cell data, but also single-cell states for many cells at multiple time points. Measurements lose meaning without context: data must be stored with metadata including detailed cell line and (molecular) growth media details, biophysical culture conditions, who performed the measurements, what instruments were used, and what software tools were used for analysis.

Current progress

Great strides have been made towards this challenge. The Open Microscopy Environment (OME) has emerged as a biological image standard with extensive metadata [9], which has helped to make scientific instruments more interoperable. The ISA-Tab format [10] functions as a rich online file system: provenance and other metadata are bundled with raw data of any file type, allowing the contents to be indexed and searched without detailed knowledge of the data formatting. This has facilitated the creation of large databases of very heterogeneous data (such as GigaDB [11]), and it enables simple data exchange due to its support for many data types.

While these formats facilitate file-level interoperability, they do not encode extracted biological data elements. Protocols.IO was developed to share detailed experimental protocols [12], which can be cited in journal publications to help improve repeatability and reproducibility. However, the protocols are human-readable checklists; they do not use a machine-readable controlled vocabulary of growth factors and other culture conditions.

Ontologies such as the Medical Subject Headings (MeSH)

[13, 14] and the Cell Behavior Ontology (CBO) can annotate many biological concepts [15], but they serve as controlled vocabularies rather than standardized data formats.

The Systems Biology Markup Language (SBML) is a well-established standard for single-cell systems biology [16], and efforts such as SBML-Dynamic are working to extend SBML to multicellular models. Domain experts in computational biology, experimental biology, and data science worked together to draft MultiCellDS, a standard for multicellular data [17]. MultiCellDS has a highly extensible representation of single-cell phenotype built from a variety of ontologies such as MeSH and CBO, which can be used to represent highly multiplex data (e.g., [1]) for many cells, along with metadata and microenvironmental context. The European Union-funded MULTIMOT project has been developing a community-driven standard for cell motility measurements (MIACME: Minimum Information about Cell Migration Experiments), with a corresponding software ecosystem [18] that can interface with data in ISA-Tab and OME formats.

Future

None of these efforts has completely addressed this challenge. Ultimately, we should combine and extend them into a unified data format. ISA-Tab could bundle image data (using OME) and extracted biological features (e.g., with MultiCellDS and MULTIMOT), while storing experimental protocol details with a controlled vocabulary growing out of Protocols.IO [12].

We must ensure that metadata not only annotate experimental protocols, but also data extraction protocols: What algorithms were used to extract the biological data elements, and where is the source code permanently archived? Some popular data science software (e.g., Docker and Jupyter notebooks) allow users to export their computational pipelines facilitate this reproducibility. Lastly, note that extracted biological data elements cannot *replace* raw data: end users must be free to reproduce (and improve!) the extraction of data elements, which requires access to the original data.

2. Shared multicellular observational representations

Beyond quantitative measurements like cell division rates, we need a machine-readable encoding of qualitative observations and insights derived from raw biological data: when cells are in condition *X*, they do *Y*. When cells of type *X* and *Y* interact by contact, they tend to do *Z*. When cell line *X* looks like *Y* in an experiment, the cell culture medium lacked factor *Z*.

Labs and clinics are replete with such examples of hard-won knowledge, but until we can systematically record them, these insights will remain siloed, isolated, and destined to be relearned, lab by lab. If we could consistently record qualitative observations, we could progress from single-cell measurements to multicellular systems understanding, including annotation of critical cell-cell interactions.

Until we can specify "correct" model behavior with machine-readable annotations, our simulation studies will be rate-limited to how quickly humans can view simulations and assess them as more or less "realistic." How do we say, in a generalized way, that a simulated tumor stays compact or becomes invasive? How do we know if a simulated developmental process has the "right" amount of branching? What does it mean for simulated image *X* to "look like" experimental image *Y*, given that both the simulation and the experiment are single instances of stochastic processes? If we cannot record the qualitative behavior of simulations and experiments, we cannot automate processes to compare them.

Current progress

Progress on this challenge has been limited. The CBO [15] has developed a good starting vocabulary for observed cell behaviors. Extensions of SBML [16] could also potentially represent some of these multicellular and multiscale observations. Tailored image processing has been applied to individual investigations to extract (generally quantitative) representations, although to date we have seen few (if any) qualitative descriptors generated by systematic image analysis.

There has been greater progress in presenting phylogenetic relationships in multicellular populations with automatically extracted phylogenetic trees and other data visualizations, such as Muller plots (e..g, [19, 20, 21]). These techniques examine large multiomics datasets (e.g., scRNA-seq data [22]) to fit and represent lineage relationships between cell types (or classes) with directed graph data structures.

Future

This area ~~seems~~ is ripe for machine learning: given a set of qualitative descriptors like "compact" versus "invasive," "mixed" versus "separated," "growing" versus "shrinking" or "steady," a neural network could be trained to human classifications of experimental and simulation data. High-throughput multicellular simulators (e.g., [6]) could create large sets of training data in standardized formats with clear ground truths. Machine vision could also be be used to analyze time series of multicellular data. These annotations could give rise to metrics that help us systematically compare the behavior of one simulation with another, or to determine which simulation (in a set of hundreds or thousands of simulations) behaves most like an experiment.

Graph structures could also be applied to represent and visualize cell-cell interactions in multicellular populations [17], similarly to phylogenetic trees (e.g., [20, 21]), chemical reaction networks (e.g., [23, 24]), gene network diagrams (e.g., [25]), and emerging data formats for agent-based model rules (e.g., as in Morpheus [26]).

3. Standards support in computational tools

For data standards to be truly useful, they must be broadly supported by a variety of interoperable tools.

Current progress

Single-cell systems biology has already shown the enabling role of stable data standards [27]: once SBML crystallized as a stable data language, a rich and growing ecosystem of data-compatible simulation and analysis software emerged. Multicellular systems biology has not yet reached this point: most computational models have custom configuration and output formats, sometimes with customized extensions of SBML to represent single-cell systems biology [17].

Future

If a multicellular data standard emerges, key open source projects [27] can implement read and write support in their software, either "natively" (i.e., at run-time), or as data converters. Hackathons or similar hosted workshops could facilitate this work. Ontologists need to provide user-friendly data bindings to simplify these development efforts. If standards are to be supported more broadly than just major open source packages, we must remember that most scientific software is created with little formal software engineering training; the data bindings must be well-documented, have simple syntax, and require minimal installation effort.

4. Shared tools to configure models and explore data

It is not enough to simply read and write data into individual tools. We must reverse the current "lock in" effect: because multicellular modeling software is difficult to learn, users (and often entire labs) focus their training on a single modeling approach. Because of this, replication studies are rare, even when a study's source code and data are openly available.

To solve this, we need user-friendly tools to import and set biological and biophysical parameters, design the virtual geometry, and write standardized configuration files that initialize many modeling frameworks. Users could run models in multiple software packages, replicate the work of others, and avoid software-specific artifacts that can bias their conclusions.

Shared software to read, analyze, compare, and visualize outputs from multiple modeling packages could reduce the learning curve for new software. If the shared data exploration and analysis tools were written to work on a common format that includes segmented experimental data, they could also be used to explore experimental data, make and annotate new observations, and motivate new model hypotheses [28].

Current progress

Without a common format for multicellular simulation data, there has been little opportunity to develop shared tools for configuring, running, and visualizing multicellular simulations. Some individual simulation packages such as Morpheus [26] and CompuCell3D [29] have user-friendly graphical model editors, but they are currently limited to their individual user communities and not compatible with other simulation packages [27]. Commercially-backed open source software such as Kitware's ParaView [30] is commonly used to visualize multicellular simulation data, but only by writing customized, simulation-tailored data importers. ParaView is generally not used to visualize biological data.

Cloud-hosted tools have provided a means to share sophisticated tools with broad, multidisciplinary audiences without need for downloading and compiling the tools. For example, the National Cancer Institute (NCI) has introduced NCI cloud resources as part of the NCI Cancer Research Data Commons [31]. Sophisticated simulation models can also be shared as web applications: the PhysiCell development team recently created xml2jupyter [32] to automatically create Jupyter-based graphical user interfaces (GUIs) for PhysiCell-based multicellular simulations, which can then be cloud-hosted on platforms like nanoHUB [33].

Other model and data sharing paradigms that emerged to address related issues in reproducibility may also encourage reuse, such as bundling data and software with Binder [34] or *GigaScience*'s recent partnership with CodeOcean to pair papers with cloud-hosted executable platforms [35]. However, these typically are single-purpose workflows (specialized to a specific data analysis for a single paper) that are not designed for modular reuse in new research workflows. They tend to lack standardized data formats (see Challenges 1-2) to facilitate connection with other tools, and latency issues will challenge their use use in high-throughput workflows. Moreover, note that while cloud-hosted executable codes increase accessibility and availability, they must not substitute for (or circumvent) sharing source code for full reproducibility.

Future

It will be difficult to make progress on this challenge without stable standards for multicellular input and output data. However, progress could be made using current draft standards, such as MultiCellDS [17]. ParaView could use customized plugins to support emerging standards for multicellular data. If projects like Morpheus implemented standards, their graphical model editors could become valuable community resources.

Hackathons can help to rapidly prototype new tools (particularly if they are paired with benchmark datasets), but they must aim to create well-documented, engineered software that are maintained in the long term. We may need new funding paradigms to support small open source teams. The form of these funding paradigms is not fully clear. Hackathons and similar forms of focused, small-team collaboration could possibly be sponsored through existing federal and philanthropic mechanisms for meetings and travel grants. Crowdsourcing could potentially fund some focused community tool development and maintenance. There is also room for creativity among funding organizations for smaller grants with faster review cycles for community tool building efforts.

Lastly, shared code platforms such as the NCI Data Commons could provide an environment to connect data and tools in online, easy-to-use workflows that encourage scientists to "mix and match" data software components into unique research. However, it will be important to avoid "lock-in" effects that prevent moving data and tools from one platform to another. Moreover, as workflows come to incorporate more web services (in differing platforms), they could become vulnerable to technical failures, business failures, or malicious attacks. Open source software has largely solved these issues by mirroring software repositories. Web services may need similar mirroring, and open science norms will need to encourage source code sharing and data/tool portability for web platforms just as they have for offline code.

5. High-quality, multiscale benchmarking datasets

Once we have standardized data formats and an ecosystem of compatible software to support them, we need high-quality datasets to drive the development of computational models. The ideal datasets would sufficiently resolve single-cell morphologies and multi-omic states in 3-D tissues, along with microenvironmental context (e.g., spatial distribution of oxygen).

To capture the behavioral states of cells, we need standard immunohistochemical panels that capture multiple dimensions of cell phenotype: cycle status, metabolism, death, motility (including markers for the leading edge), adhesiveness, cell mechanics, polarization, and more. We will need to capture these details simultaneously in many cells at multiple time points, using massively multiplexed technologies.

These datasets would be used to formulate model hypotheses and assumptions (through data exploration using standardized tools), to train models, and to evaluate them. Moreover, as the community develops new computational models, they could be evaluated against benchmark datasets. Benchmark datasets are domain-specific: separate datasets are needed for developmental biology, avascular and vascular tumor growth, autoimmune diseases, and other problems. It is important that these datasets are easily accessible with open data licenses to promote the broadest use possible. Adhering to FAIR (Findability, Accessibility, Interoperability, and Reusability) data principles would be ideal [36].

Current progress

Cancer biology has made perhaps the greatest progress on this challenge, where the NIH-funded Cancer Genome Atlas hosts many genomic, microscopy, and other large datasets [37]. Typically, these consist of many samples at a single time, rather than time course data. Highly multiplex multicellular data are generally not available. DREAM challenges have assembled high-quality datasets to drive model development (through competitions) [38], but these have not typically satisfied the multiplex, time series ideals outlined above. Private founda-

tions are using cutting-edge microscopy to create high-quality online datasets (e.g., the Allen Cell Explorer Project [39]).

The technology for highly-multiplexed measurements is steadily improving: CyTOF-based immunohistochemistry (e.g., as in Levenson et al. [1]) can stain for panels of 30-50 immunomarkers on single slides at 1-2 μm resolution or better. There are no standardized panels to capture the gamut of phenotypic behaviors outlined above. Social media discussions (e.g., [40]) have helped to drive community dialog on difficult phenotypic parameters, but no clear consensus has emerged for a "gold standard" panel of immunostains.

Future

Workshops of leading biologists should assemble the "dream panel" of molecular markers. Consortia of technologists will need to reliably implement these multi-parameter panels in experimental workflows [1]. Workshops of bioinformaticians, data scientists, and modelers will be needed to "transform" these raw data into standardized datasets for use in models. All this will require federal or philanthropic funding, and contributions by multiple labs. Social media has great potential for public brainstorming, disseminating resources, and recruiting new contributors. Hackathons could help drive the "translation" of raw image data into standardized datasets, while developing tools that automate the process.

6. Community-curated public data libraries

We need "public data libraries" to store and share high-quality, standardized data [41, 28]. Data should not be static: the community should continually update data to reflect scientific advances, with community curation to ensure data quality. Public libraries must not only store raw image data and extracted biological parameters, but also qualitative observations and human insights. The public libraries should host data at multiple stages of publication: preliminary data (which may or may not be permanently archived), datasets under construction (i.e., the experiments are ongoing), data associated with a preprint or a paper in review, and data associated with a published work. Public data libraries should enable if not encourage versioned post-publication refinement, particularly for datasets arising from secondary analysis or curation of heterogeneously sourced primary raw data, such as digital cell lines [17]. Lastly, public data libraries need to be truly public by using licenses (e.g., Creative Commons CC0 or CC-BY) that encourage new derivative works, as well as aggregation into larger datasets.

Current progress

Numerous data portals exist, and more are emerging. Many are purpose-built for specific communities, such as the Cancer Genome Atlas [37]. The Image Data Resource [42] was recently launched to facilitate sharing bioimages using the OME data format [9], further demonstrating how standardized data can facilitate the creation of shared tools and resources. Others like GigaDB [11] and DRYAD [43] allow users to post self-standing datasets with unique DOIs to facilitate data reuse and attribution. These repositories are free for access, thus increasing the reach and impact of hosted data, but the data contributors must pay at the time of data publication. The fees often include editorial and technical assistance while ensuring long-term data availability.

Even within single data hosting repositories, individual datasets are largely disconnected and mutually non-interoperable beyond ISA-Tab compatibility. Thus, individual hosted datasets and studies are generally not bridged and recombined. Moreover, the datasets are usually static after publication, rather than actively curated and updated. BioNumbers has long served as a searchable resource of user-contributed biological parameters [44], but it lacks a unified data model. The MultiCellDS project proposed *digital cell lines*, which aggregate measurements from many sources for a single cell type [17]. Digital cell lines were intended to be continually updated and curated by the community, so that low-quality measurements could be replaced by better measurements as technology advances. However, this effort is currently manual, with no single, easily searchable repository for its pilot data.

An unfortunate consequence of the current data hosting model is that all the burden rests on data donors: they generate the data, format it to standards, assemble it, document it, upload it, and then pay the hosting and scientific publication costs. This is a classic case of the "*tragedy of the commons*": it is easy to benefit from shared resources, but the cost of contribution falls on contributors. ~~costly to contribute.~~ Most repositories have fee waivers for scientists in low-income nations, but small and underfunded labs and citizen scientists are still at a disadvantage.

Nonprofit organizations like DRYAD have made great strides in creating sustainable resources to host data; currently (as of 2019), a one-time charge of US $120 per dataset applies once the data are accepted by curators and publicly available [43]. This is a small fee compared to the data generation cost for experimental labs and within the means of well-funded labs. In cases where secondary analyses or simulations generate new datasets independent of grant funding, there may be greater hardship in these costs, particularly when coupled with open access publication fees.

Future

We need to develop more unified, ~~financially stable and~~ scalable repositories that can bridge fields and collect our knowledge. The repositories should ~~need to~~ be indexed and community curated ~~and continually improved, rather than static~~ to encourage continuous refinement where possible. While there has been great progress to create financially sustainable, permanent data hosting, there is still room to explore alternative funding for data generated independently of specific grant funds. Moreover, these archive-oriented data stores still require curation and indexing if they are to grow from data storage to libraries.

Solutions to this challenge may well originate outside the bioinformatics community. Library scientists have longstanding domain expertise in collecting and curating knowledge across disciplines in unified physical libraries: this expertise would undoubtedly benefit any efforts to create public data libraries. The tremendous success of Wikipedia [45] in hosting its own image and video resources on Wikimedia Commons [46]—at no cost to contributors—could be a very good model. bioRxiv [47] has been similarly successful in hosting preprints at no cost to authors, although experimental data hosting costs are far higher than the cost of hosting manuscripts. Both of these have relied upon a combination of public donations, federal support, and philanthropy, channeled through appropriate nonprofit structures.

We note that public data libraries could become victims of their own success: as public repositories proliferate, finding information will become increasingly difficult, and the community of contributors could become fragmented. This, in turn, will make it difficult to recruit data curators to maintain the quality of the resources. Thus, the community may need to reach consensus on which libraries serve as the standard repositories for which types of data. Moreover, unified search engines and indices may be needed to help unify knowledge in existing and new data libraries.

Lastly, to ensure robustness and sustainability, we need to encourage data mirroring with global searchability, and pro-

mote a culture that values and properly cites all contributions to shared knowledge: data generation, data analysis, and data curation. While badges can help [48, 49], we must ensure that data users can easily cite all these contributions in papers, that impact metrics reflect the breadth of contributions, and that tenure and other career processes truly value all contributions to community knowledge resources.

7. Quality and curation standards

Community-curated public libraries face new questions: how can we consistently decide which data are worth saving? How do we determine if a new measurement is better than an old one? How do we monitor quality? Can we automatically trust one lab's data contributions based upon prior contributions? And who gets to make these decisions?

Current progress

Little to none, aside from uncertainty quantification.

Future

This challenge is as much cultural as it is technical. We will need to hold workshops of leading biologists to identify community values and standards for assessing different measurement types. The community will need to determine if "gold standards" can be devised for comparing measurements.

8. Linking data to models

We need to connect data to computational models. Data modelers should help design experiments, to determine what variables are needed to build useful models. We need to determine how to "map" biological measurements to model parameters.

Current progress

This challenge is currently being addressed on a study-by-study basis. Individual teams design experiments, devise their own model calibration methods, formulate model evaluation metrics, and create their own tools to analyze and compare experimental and simulation data.

Future

This challenge is both technical and cultural. Mathematicians, biologists, data scientists, and others will need to work together to determine what it means for an inherently stochastic simulation model to match to match an experiment. Any progress in creating standardized data elements and annotating multicellular systems behaviors will surely help in creating metrics to compare experimental and computational models. Once standardized biological parameters are extracted to create benchmark datasets, machine learning could help drive more systematic mappings from extracted biological parameters to computational model inputs.

Conclusions

The time is ripe for data-driven multicellular systems biology and engineering. Technological advances are making it possible to create high-resolution, highly multiplex multicellular datasets. Computational modeling platforms—including simulation and machine learning approaches—have advanced considerably, and they are increasingly available as open source [27, 50]. Supercomputing resources are amplifying the power of these computational models [6, 7], while cloud resources are making them accessible to all [33, 32].

If we can solve these key challenges, we will connect big multicellular datasets with computational technologies to accelerate our understanding of biological systems. Steady, incremental progress towards any of the challenges benefit the community as we move towards this broader vision.

Some of the challenges are largely technical, such as creating data standards. Others are more cultural, such as shaping community values for data curation. All of the challenges share a need for community investment: developing and sharing compatible tools and data, hosting data, curating public data libraries, and ultimately funding these worthwhile efforts. Many groups are already contributing pieces of this puzzle, often with little financial support. In the future, we must reduce the individual burden in creating community goods. We may need newer, more rapid funding paradigms to help support and harden new software tools, scaling from small but simple proposals to the current large software grant mechanisms (which tend to have low funding rates). We may need to fund software labs rather than software projects, to encourage rapid response to emerging community needs.

We are on the cusp of accelerated, data-driven biological discovery of how cells work together, how they build things, and how this breaks to cause disease. If you are working towards solving any of these challenges (or if you have new ones to pose!), please consider sharing your advances here.

Declarations

List of abbreviations

**CBO**	Cell Behavior Ontology
**CC0**	Creative Commons public domain license
**CC-BY**	Creative Commons attribution license
**CyTOF**	Cytometry by Time Of Flight
**DOI**	Digital Object Identifier
**DREAM**	Dialogue for Reverse Engineering Assessments and Methods
**FAIR**	Findability, Accessibility, Interoperability, and Reusability
**ISA-Tab**	Investigation-Study-Assay tabular format
**MeSH**	Medical Subject Headings
**NIH**	(U.S.) National Institutes of Health
**OME**	Open Microscopy Environment
**SBML**	Systems Biology Markup Language

Competing Interests

The author declares that he has no competing interests.

Funding

PM's work to develop computational tools and data standards for multicellular systems biology was funded by the Breast Cancer Research Foundation (PIs Agus, Ewald, Gilkes and Macklin) and the Jayne Koskinas Ted Giovanis Foundation for Health and Policy (PIs Ewald, Gilkes, and Macklin), the National Science Foundation (PI Fox, 1720625), and the National Cancer Institute (PIs Finley, Macklin, and Mumenthaler, U01-CA232137-01; PIs Agus, Atala, and Soker, 1R01CA180149; PI Hillis, 5U54CA143907).

Author's Contributions

PM conceptualized and wrote the manuscript.

Acknowledgements

PM thanks Nicole Nogoy and the staff of *GigaScience* for the opportunity to write this review, and for editorial support. PM thanks the reviewers and preprint readers for valuable comments and feedback.

Author's information

PM has worked for over ten years in computational multicellular systems biology, with a focus on cancer biology and tissue engineering. He has written several open source tools for the field, including BioFVM (a multi-substrate diffusion solver for biochemical cell-cell communication) [51], PhysiCell (a 3-D agent-based modeling toolkit) [52], and MultiCellDS (a draft multicellular data standard) [17]. He is an Associate Professor of Intelligent Systems Engineering at Indiana University.

References

1. Levenson RM, Borowsky AD, Angelo M. Immunohistochemistry and mass spectrometry for highly multiplexed cellular molecular imaging. Laboratory Investigation 2015;95:397. https://dx.doi.org/10.1038/labinvest.2015.2.
2. Kim M, Rai N, Zorraquino V, Tagkopoulos I. Multi-omics integration accurately predicts cellular state in unexplored conditions for Escherichia coli. Nat Comm 2016;7:13090. http://dx.doi.org/10.1038/ncomms13090.
3. Norris JL, Farrow MA, Gutierrez DB, Palmer LD, Muszynski N, Sherrod SD, et al. Integrated, High-Throughput, Multiomics Platform Enables Data-Driven Construction of Cellular Responses and Reveals Global Drug Mechanisms of Action. J Proteome Res 2017;16(3):1364–1375. https://doi.org/10.1021/acs.jproteome.6b01004.
4. Adli M. The CRISPR tool kit for genome editing and beyond. Nat Comm 2018;9(1):1–13. https://doi.org/10.1038/s41467-018-04252-2.
5. Blundell JR, Levy SF. Beyond genome sequencing: Lineage tracking with barcodes to study the dynamics of evolution, infection, and cancer. Genomics 2014;104(6, Part A):417 – 430. http://www.sciencedirect.com/science/article/pii/S0888754314001827, experimental evolution and the use of genomics.
6. Ozik J, Collier N, Wozniak J, Macal C, Cockrell C, Friedman SH, et al. High-throughput cancer hypothesis testing with an integrated PhysiCell-EMEWS workflow. BMC Bioinformatics 2018;19(Suppl 18):483. http://dx.doi.org/10.1186/s12859-018-2510-x.
7. Ozik J, Collier N, Heiland R, An G, Macklin P. Learning-accelerated Discovery of Immune-Tumour Interactions. Molec Syst Design Eng 2019;4:747–60. http://dx.doi.org/10.1039/c9me00036d.
8. Macklin P. When Seeing Isn't Believing: How Math Can Guide Our Interpretation of Measurements and Experiments. Cell Sys 2017;5(2):92–4. http://dx.doi.org/10.1016/j.cels.2017.08.005.
9. Goldberg IG, Allan C, Burel JM, Creager D, Falconi A, Hochheiser H, et al. The Open Microscopy Environment (OME) Data Model and XML file: open tools for informatics and quantitative analysis in biological imaging. Genome Biology 2005 May;6(5):R47. https://doi.org/10.1186/gb-2005-6-5-r47.
10. Rocca-Serra P, Brandizi M, Maguire E, Sklyar N, Taylor C, Begley K, et al. ISA software suite: supporting standards-compliant experimental annotation and enabling curation at the community level. Bioinformatics 2010;26(18):2354–2356. http://dx.doi.org/10.1093/bioinformatics/btq415.
11. GigaDB Website; visited 2018-05-17. http://gigadb.org.
12. Teytelman L, Stoliartchouk A, Kindler L, Hurwitz BL. Protocols.io: Virtual Communities for Protocol Development and Discussion. PLOS Biology 2016 08;14(8):1–6. https://doi.org/10.1371/journal.pbio.1002538.
13. Rogers FB. Communications to the Editor. Bull Med Lib Assoc 1963;51(1):114–6. https://www.ncbi.nlm.nih.gov/pmc/articles/PMC197951/.
14. Lipscomb CE. Medical Subject Headings (MeSH). Bull Med Lib Assoc 2000;88(3):265–6. https://www.ncbi.nlm.nih.gov/pmc/articles/PMC35238/.
15. Sluka JP, Shirinifard A, Swat M, Cosmanescu A, Heiland RW, Glazier JA. The cell behavior ontology: describing the intrinsic biological behaviors of real and model cells seen as active agents. Bioinformatics 2014;30(16):2367–2374. http://dx.doi.org/10.1093/bioinformatics/btu210.
16. Hucka M, Finney A, Sauro HM, Bolouri H, Doyle JC, Kitano H, et al. The systems biology markup language (SBML): a medium for representation and exchange of biochemical network models. Bioinformatics 2003;19(4):524–531. http://dx.doi.org/10.1093/bioinformatics/btg015.
17. Friedman SH, Anderson ARA, Bortz DM, Fletcher AG, Frieboes HB, Ghaffarizadeh A, et al. MultiCellDS: a community-developed standard for curating microenvironment-dependent multicellular data. bioRxiv 2016;090456. http://dx.doi.org/10.1101/090456, (**Preprint**, preparing for resubmission).
18. Masuzzo P, Martens L. An open data ecosystem for cell migration research. Trends Cell Biol 2015;25(2):55–8. https://doi.org/10.1016/j.tcb.2014.11.005.
19. Qiu P, Simonds EF, Bendall SC, Gibbs KD, Bruggner RV, Linderman MD, et al. Extracting a cellular hierarchy from high-dimensional cytometry data with SPADE. Nat Biotech 2011;29(10):886–91. https://dx.doi.org/10.1038/nbt.1991.
20. Hicks DG, Speed TP, Yassin M, Russell SM. Maps of variability in cell lineage trees. PLOS Computational Biology 2019 02;15(2):1–32. https://doi.org/10.1371/journal.pcbi.1006745.
21. Gatenbee CD, Schenck RO, Bravo R, Anderson ARA. EvoFreq: Visualization of the Evolutionary Frequencies of Sequence and Model Data. bioRxiv 2019;https://www.biorxiv.org/content/early/2019/08/22/743815.
22. Satija R, Farrell JA, Gennert D, Schier AF, Regev A. Spatial reconstruction of single-cell gene expression. Nat Biotech 2015;33(5):495–502. https://www.ncbi.nlm.nih.gov/pmc/articles/PMC4430369/.
23. Blinov ML, Yang J, Faeder JR, Hlavacek WS. Graph Theory for Rule-Based Modeling of Biochemical Networks. In: Priami C, Ingólfsdóttir A, Mishra B, Riis Nielson H, editors. Transactions on Computational Systems Biology VII Berlin, Heidelberg: Springer Berlin Heidelberg; 2006. p. 89–106.
24. Lambusch F, Waltemath D, Wolkenhauer O, Sandkuhl K, Rosenke C, Henkel R. Identifying frequent patterns in biochemical reaction networks: a workflow. Database 2018 07;2018. https://doi.org/10.1093/database/bay051, bay051.
25. Zhang M, Li Q, Yu D, Yao B, Guo W, Xie Y, et al. GeNeCK: a web server for gene network construction and visualization. BMC Bioinformatics 2019;20(1):12. https://doi.org/10.1186/s12859-018-2560-0.
26. Starruß J, de Back W, Brusch L, Deutsch A. Morpheus: a user-friendly modeling environment for multiscale and multicellular systems biology. Bioinformatics 2014;30(9):1331–1332. http://dx.doi.org/10.1093/bioinformatics/btt772.

27. Macklin P, Friedman SH, MultiCellDS. Open source tools and standardized data in cancer systems biology. bioRxiv 2018;244319. https://dx.doi.org/10.1101/244319, (part of the NCI CSBC/PS-ON Handbook of Mathematical Oncology).
28. Zaritsky A. Sharing and reusing cell image data. Molecular Biology of the Cell 2018;29(11):1274–1280. https://doi.org/10.1091/mbc.E17-10-0606.
29. Swat MH, Thomas GL, Belmonte JM, Shirinifard A, Hmeljak D, Glazier JA. Chapter 13 - Multi-Scale Modeling of Tissues Using CompuCell3D. In: Asthagiri AR, Arkin AP, editors. Computational Methods in Cell Biology, vol. 110 of Methods in Cell Biology Academic Press; 2012.p. 325 – 366. http://dx.doi.org/10.1016/B978-0-12-388403-9.00013-8.
30. Kitware, ParaView Website; visited 2018-05-17. https://www.paraview.org/.
31. NCI Cloud Resources; visited 2019-09-26. https://datascience.cancer.gov/data-commons/cloud-resources.
32. Heiland R, Mishler D, Zhang T, Bower E, Macklin P. xml2jupyter: Mapping parameters between XML and Jupyter widgets. Journal of Open Source Software 2019;4(39):1408. http://dx.doi.org/10.21105/joss.01408.
33. Madhavan K, Zentner L, Farnsworth V, Shivarajapura S, Zentner M, Denny N, et al. nanoHUB.org: cloud-based services for nanoscale modeling, simulation, and education. Nanotech Rev 2013;2(1):107–17. https://doi.org/10.1515/ntrev-2012-0043.
34. Toward publishing reproducible computation with Binder; visited 2019-09-26. https://elifesciences.org/labs/a7d53a88/toward-publishing-reproducible-computation-with-binder.
35. Data Intensive Software Publishing & Sailing The Code Ocean. Q&A With Ruibang Luo; visited 2019-09-26. http://gigasciencejournal.com/blog/data-intensive-software-publishing-sailing-the-code-ocean-qa-with-ruibang-luo/.
36. Wilkinson MD, Dumontier M, Aalbersberg IJ, Appleton G, Axton M, Baak A, et al. The FAIR Guiding Principles for scientific data management and stewardship. Scientific Data 2016;3:160018. http://dx.doi.org/10.1038/sdata.2016.18.
37. The Cancer Genome Atlas; visited 2018-05-17. https://cancergenome.nih.gov/.
38. DREAM Challenges Project Website; visited 2018-05-17. http://dreamchallenges.org/.
39. Allen Institute, Allen Cell Explorer Project; visited 2018-05-17. http://www.allencell.org/cell-catalog.html.
40. @MAG2ART, @MathCancer, @Alexis_Lomakin, @KidneyMyosin, Twitter discussion on cell motility immunostains; visited 2017-12-13. https://twitter.com/MAG2ART/status/940942422074384384.
41. Ellenberg J, Swedlow JR, Barlow M, Cook CE, Sarkans U, Patwardhan A, et al. A call for public archives for biological image data. Nat Meth 2018;15(11):849–54. https://doi.org/10.1038/s41592-018-0195-8.
42. Williams E, Moore J, Li SW, Rustici G, Tarkowska A, Chessel A, et al. Image Data Resource: a bioimage data integration and publication platform. Nat Meth 2017;14:775–81. https://doi.org/10.1038/nmeth.4326.
43. DRYAD Digital Repository; visited 2018-05-17. https://datadryad.org/.
44. Milo R, Jorgensen P, Moran U, Weber G, Springer M. BioNumbers—the database of key numbers in molecular and cell biology. Nucleic Acids Research 2010;38(Suppl. 1):D750–D753. http://dx.doi.org/10.1093/nar/gkp889.
45. Wikipedia; visited 2018-05-17. https://wikipedia.org.
46. Wikimedia Commons; visited 2018-05-17. https://www.wikimedia.org/.
47. bioRxiv: The Preprint Server for Biology; visited 2018-05-17. https://biorxiv.org.
48. Kidwell MC, Lazarević LB, Baranski E, Hardwicke TE, Piechowski S, Falkenberg LS, et al. Badges to Acknowledge Open Practices: A Simple, Low-Cost, Effective Method for Increasing Transparency. PLOS Biology 2016 05;14(5):1–15. https://doi.org/10.1371/journal.pbio.1002456.
49. Rowhani-Farid A, Allen M, Barnett AG. What incentives increase data sharing in health and medical research? A systematic review. Research Integrity and Peer Review 2017 May;2(1):4. https://doi.org/10.1186/s41073-017-0028-9.
50. Macklin P, Frieboes HB, Sparks JL, Ghaffarizadeh A, Friedman SH, Juarez EF, et al. Progress Towards Computational 3-D Multicellular Systems Biology. In: Rejniak KA, editor. Systems Biology of Tumor Microenvironment Springer; 2016.p. 225–46. (invited author: P. Macklin).
51. Ghaffarizadeh A, Friedman SH, Macklin P. BioFVM: an efficient, parallelized diffusive transport solver for 3-D biological simulations. Bioinformatics 2016;32(8):1256–8. http://dx.doi.org/10.1093/bioinformatics/btv730.
52. Ghaffarizadeh A, Heiland R, Friedman SH, Mumenthaler SM, Macklin P. PhysiCell: an open source physics-based cell simulator for 3-D multicellular systems. PLoS Comput Biol 2018;14(2):e1005991. http://dx.doi.org/10.1371/journal.pcbi.1005991.

Click here to access/download;Manuscript;main.tex
Click here to view linked References

```
This is pdfTeX, Version 3.14159265-2.6-1.40.19 (TeX Live 2018/W32TeX)
(preloaded format=pdflatex 2018.7.12)  27 SEP 2019 12:47
entering extended mode
 restricted \write18 enabled.
 %&-line parsing enabled.
**main.tex
(./main.tex
LaTeX2e <2018-04-01> patch level 5

! LaTeX Error: File `oup-contemporary.cls' not found.

Type X to quit or <RETURN> to proceed,
or enter new name. (Default extension: cls)

Enter file name:
! Emergency stop.
<read *>

l.11 ^^M

*** (cannot \read from terminal in nonstop modes)


Here is how much of TeX's memory you used:
 10 strings out of 492646
 215 string characters out of 6133325
 56709 words of memory out of 5000000
 3994 multiletter control sequences out of 15000+600000
 3640 words of font info for 14 fonts, out of 8000000 for 9000
 1141 hyphenation exceptions out of 8191
 10i,0n,8p,86b,8s stack positions out of 5000i,500n,10000p,200000b,80000s
!  ==> Fatal error occurred, no output PDF file produced!
```

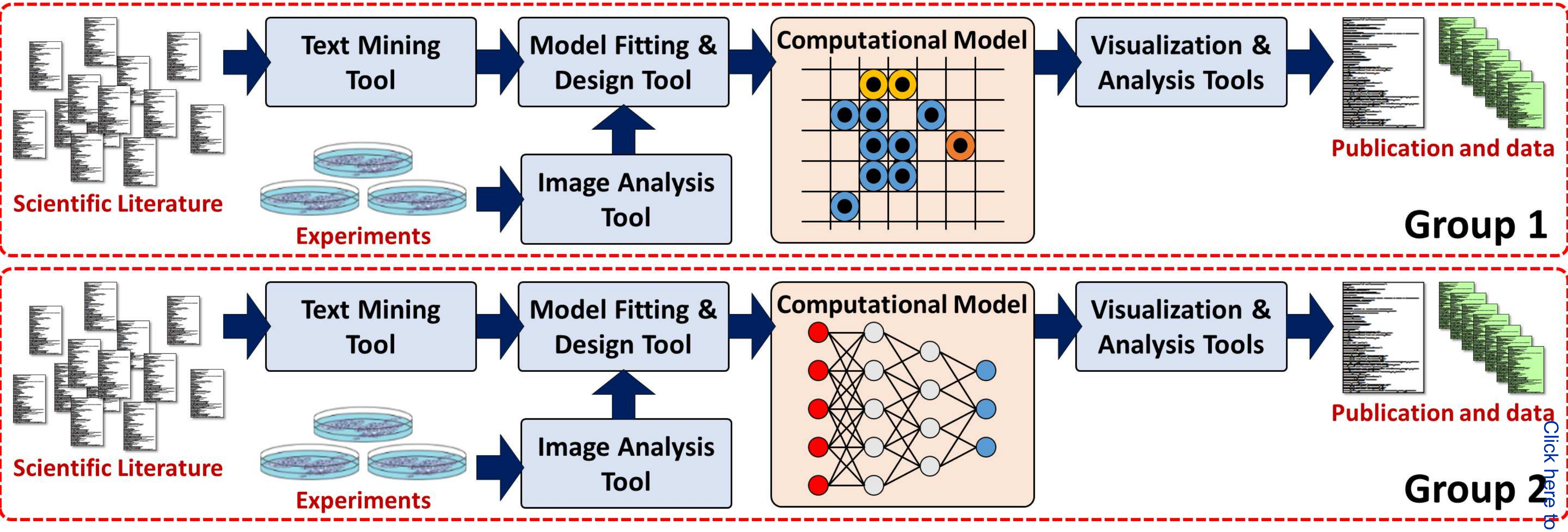

Click here to access/download;Figure;Fig1.eps

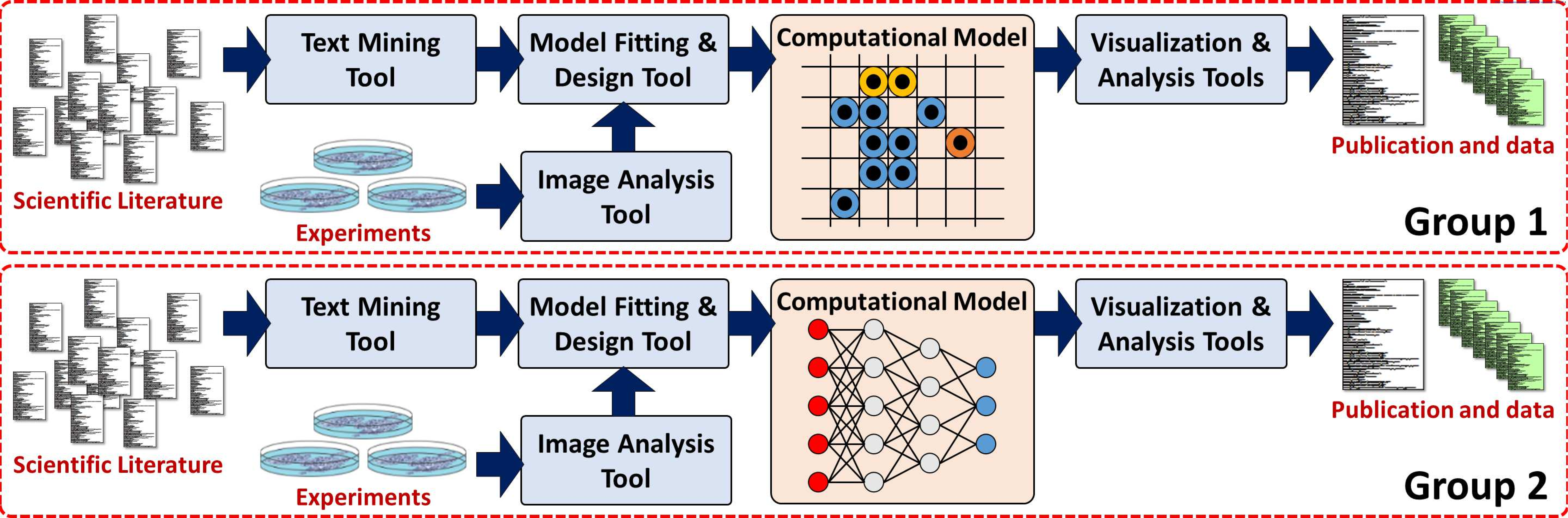
Text Mining Tool
Model Fitting & Design Tool
Computational Model
Visualization & Analysis Tools
Publication and data
Image Analysis Tool
Scientific Literature
Experiments
Group 1
Text Mining Tool
Model Fitting & Design Tool
Computational Model
Visualization & Analysis Tools
Publication and data
Image Analysis Tool
Scientific Literature
Experiments
Group 2

Click here to access/download;Figure;Fig2.eps

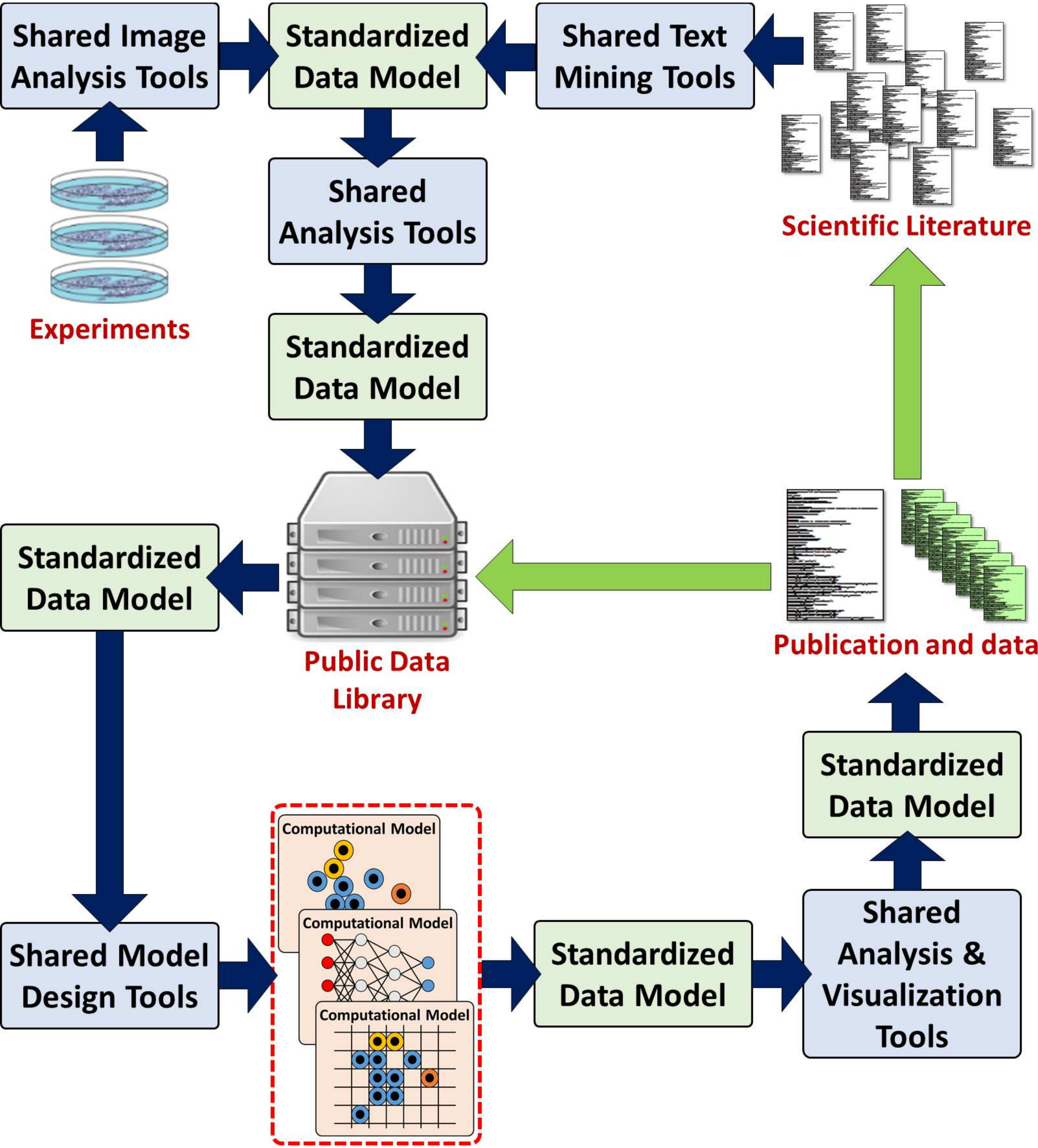

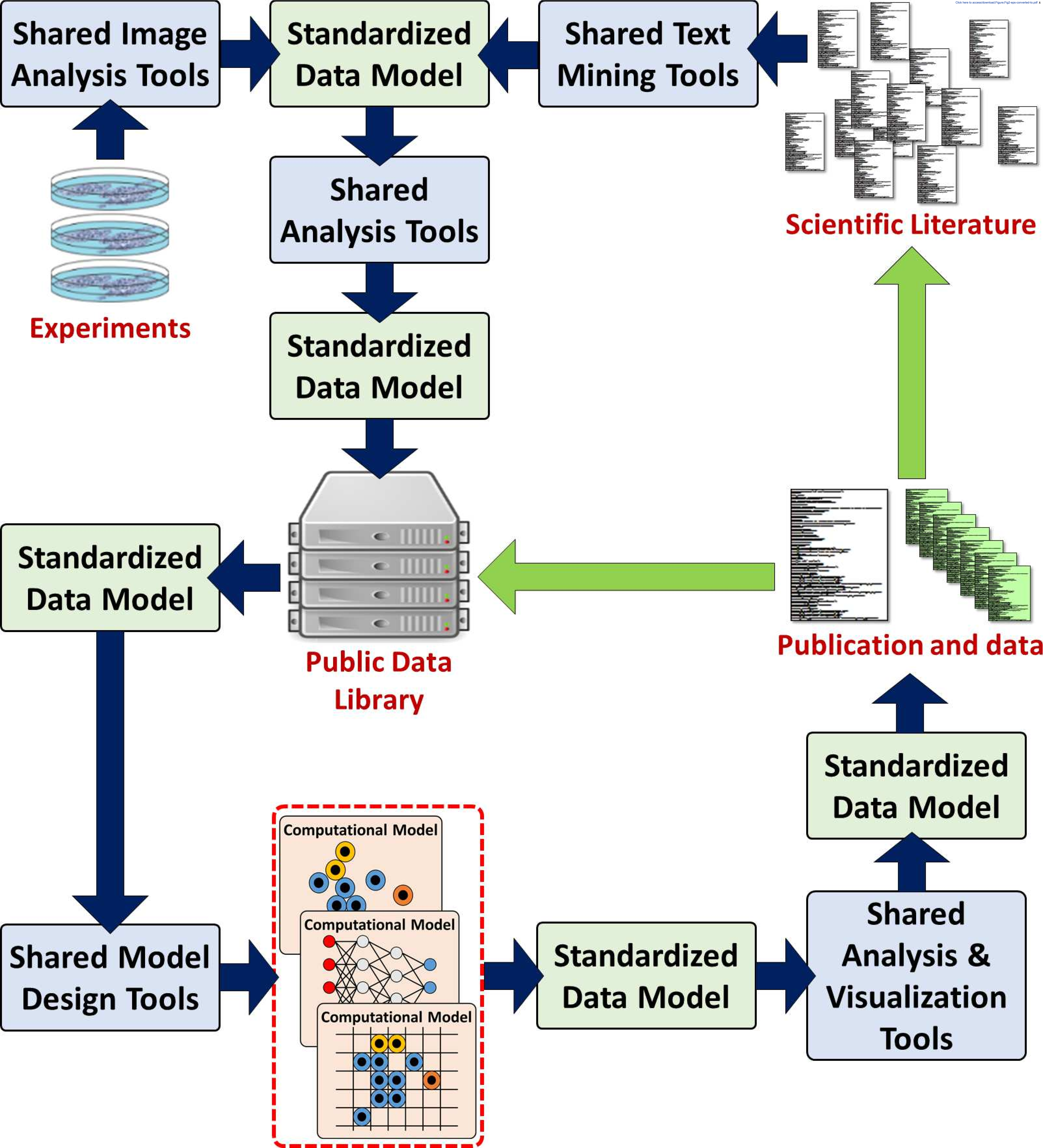

Shared Image Analysis Tools
Standardized Data Model
Shared Text Mining Tools
Experiments
Shared Analysis Tools
Scientific Literature
Standardized Data Model
Standardized Data Model
Public Data Library
Publication and data
Standardized Data Model
Computational Model
Computational Model
Computational Model
Shared Model Design Tools
Standardized Data Model
Shared Analysis & Visualization Tools

(GIGA)n SCIENCE

Click here

C
li

OXFORD

clean manuscript

Click here to access/download
Supplementary Material
main-clean.pdf

Click here to access/download
Supplementary Material
main-clean.tex

Click here to access/download
Supplementary Material
oup-contemporary.cls

Click here to access/download
Supplementary Material
paper-refs.bib

Click here to access/download
Supplementary Material
vancouver-authoryear.bst

response to reviewers Click here to access/download;Personal Cover;Resubmission-response-to-reviewers.pdf

SCHOOL OF INFORMATICS,
COMPUTING, AND ENGINEERING

INDIANA UNIVERSITY

September 27, 2019

GigaScience editorial staff

Re: Resubmission for GIGA-D-18-00182

Dear Editors:

I thank the reviewers for their constructive comments, all of which improved this manuscript. I have addressed all the critiques as detailed below. In the manuscript, changes are marked in red. (A clean version of the revised manuscript is also attached as supplementary material, as are the LaTeX document source files.) In addition to the two peer reviewers, I have also addressed emailed feedback from two preprint readers.

I believe that after having addressed these four sets of feedback, you may find this manuscript ready for publication.

Thank you for this opportunity to connect with the *GigaScience* community.

Sincerely,

Paul Macklin, Ph.D.
Associate Professor, Intelligent Systems Engineering
Director of Undergraduate Studies, Intelligent Systems Engineering
Founder & Lead, PhysiCell and MultiCellDS Projects
Member, IU Simon Cancer Center
Indiana University – Bloomington

Reviewer 1: (Brett Beaulieu-Jones)

This review discusses core issues data-driven multicellular biology faces, given rapid advances in the field (high-throughput measurement/manipulation technologies and more complex modelling). It raises questions around reproducibility, data sharing and interoperability to encourage collaboration as well as how to structure what is learned. These are unsolved problems in many fields and especially important in data-driven multicellular biology given it's complexity of experiments and data.

>> Thank you for this comment. I'm happy that you agree on the timeliness of the article and the problem it addresses.

Within the Key Challenges, it states: "We need to move beyond shared drives of raw images and spreadsheets to extracted biological elements that are useful for building models and machine learning." I would argue that this is insufficient without also including the raw data and detailed metadata explaining how biological elements are extracted. Tools and technologies (and annotations) change rapidly over time which can lead to significant differences in extracted data.

>> I appreciate and agree with this comment. When we worked on MultiCellDS, we went to great lengths to create metadata elements that preserve provenance so that future scientists and readers can always return to original source materials.

I revised to mention this important point more explicitly in the "future" part of the challenge (new text in red):

> … while storing experimental protocol details with a controlled vocabulary growing out of Protocols.IO.
>
> Metadata should not only annotate experimental protocols, but also data extraction protocols: What algorithms were used to extract the biological data elements, and where is the source code permanently archived? Some popular data science software (e.g., Docker and Jupyter notebooks) allow
>
> users to export their computational pipelines facilitate this reproducibility. Lastly, note that extracted biological data elements cannot *replace* raw data: end users must be free to reproduce (and improve!) the extraction of data elements, which requires access to the original data.

In addition to protocols.io I would also mention some of the tools that allow you to export a computational pipeline to ensure others could reproduce your (via docker, jupyter notebooks etc.).

>> Thanks! I added this great point to the section (see above)

Under shared multicellular representations: An interesting parallel would be some of the tools to extract genetic associations and/or relationships from literature. These are commonly stored in graph databases.

>> That's a really great point: multicellular interactions could definitely be represented as a directed graph structure. (Perhaps you can submit a solution to this problem!)

I referenced this great point in the "current progress" section of the challenge (new text in red):

> … by systematic image analysis.
>
> There has been greater progress in presenting phylogenetic relationships in multicellular populations with automatically extracted phylogenetic trees and other data visualizations, such as Muller plots (e.g., [refs]). These techniques examine large multiomics datasets (e.g., scRNA-seq [ref] data) to fit and represent lineage relationships between cell types (or classes) with directed graph data structures.

I also mentioned this in the "future" section of the challenge (new text in red):

> Graph structures could also be applied to represent and visualize cell-cell interactions in multicellular populations [ref], similarly to phylogenetic trees (e.g., [refs]), chemical reaction networks (e.g., [ref]), gene network diagrams (e.g., [ref]), and emerging data formats for agent-based model rules (e.g., as in Morpheus [ref]).

Under community-curated public data libraries: A final point is the idea that if we create many commons, nothing is common between them. By that I mean, it becomes hard to know where to go for what. I think it'd be helpful to further emphasize the need standards and community agreement around which data libraries serve as standards.

Paul Macklin, Ph.D. • **web**: MathCancer.org • **mobile**: 626-372-1203 • **Twitter**: @MathCancer • macklinp@iu.edu
Dept. of Intelligent Systems Engineering • Indiana University • Luddy Hall 4128, 700 N. Woodlawn Ave, Bloomington, IN 47408

>> This is a really great point. We will make a point that the proliferation of repositories itself can be a key challenge. I made the following addition to the "future" part of the challenge (new text in red):

> ... new data libraries.
>
> We note that public data libraries could become victims of their own success: as public repositories proliferate, finding information will become increasingly difficult, and the community of contributors could become fragmented. This, in turn, will make it difficult to recruit data curators to maintain the quality of the resources. Thus, the community may need to reach consensus on which libraries serve as the standard repositories for which types of data. Moreover, unified search engines and indices may be needed to help unify knowledge in existing and new data libraries.

Overall, this is a nice review of some of the challenges facing a fast-moving field that is of growing importance.

>> Thank you!

Reviewer 2: (Lenny Teytelman)

This is a review of "Key challenges facing data-driven multicellular systems biology".

My most important note is that I am a very imperfect reviewer for it. I am no expert in systems biology and as a yeast geneticist, I am certainly no guru in multicellular systems and challenges. Therefore, while I very much enjoyed the paper, I hope that others have reviewed it who are more familiar with this research topic.

Below are some suggestions that the author might want to consider.

>> Thank you for your careful review and insights. I've worked to integrate your comments, as well as those of the other reviewer plus two outside preprint readers who provided further comments.

1. The paper describes 8 "Key challenges" facing the field. Are all of these equal barriers to progress or are some more serious than others? Ranking these and highlighting which of them are indeed critical to address would be useful. As is, a reader might conclude that true progress in the field is hopeless. Moreover, if the paper is meant to serve as a call to action for funders and the community, it would be beneficial to opine in the discussion section which challenges deserve the most funding/attention. If we had to pick one of these eight to tackle, would working towards standards be most important or is focus on developing good benchmarking datasets a better first target?

>> I think all these challenges need to be addressed for the field to make its fullest impact, but I find it problematic to rank them along a single axis. Each challenge affects different aspects of work, from pragmatic / technical concerns like "how do we write data down" to "where do we store data?"

>> Moreover, I'm more of a "glass half full" type of person: each of these challenges is an opportunity to make an impact. Progress on any one of them would improve the current state. Each could potentially be addressed on its own. (e.g., we can make data repositories for unstructured data, even while figuring out data standards.) So, I prefer to not impose a one-dimensional ranking on a multi-dimensional set of problems.

>> Addressing your last statement, there is no compelling reason that one group couldn't work on a benchmark dataset (and record raw data) while another works on data standards that could later be applied to that data. The community is large, and there is no reason to work on them sequentially. I believe that all challenges deserve attention, and indeed, I hope for a "call to arms." I personally would like to see data standardizations prioritized earlier to help guide creation of datasets, but I think prioritizations ought to be a community discussion.

>> I also agree that the readers need to be encouraged to contribute without feeling daunted, or that progress cannot be made without fully solving all these challenges. In the introduction, I made the following changes to better reach this tone, and then to further address your excellent points (changes in red):

In this review, we will explore some key challenges that we need to ~~must be~~ overcome before we can reach the full potential of ~~create~~ an ecosystem of interoperable data and tools for multicellular systems biology.

While the challenges are not presented in any ranked order of importance or priority, they progress from the concrete topics of standardized data representation and knowledge capture to community resources we could build with standardized data. We do not need to address these challenges sequentially. Indeed, one of the great strengths of open research communities is that progress can occur by many groups in parallel, each contributing according to their individual skills, resources, and interests.

>> I also (manually) numbered the challenges for easier cross-referencing.

>> In the conclusions section (changes in red):

If we can solve these key challenges, we will connect big multicellular datasets with computational technologies to accelerate our understanding of biological systems. Steady, incremental progress towards any of the challenges benefit the community as we progress towards this broader vision.

2. The section "Shared tools to configure models and explore data" ends with, "We may need new funding paradigms to support small open source teams." I find this recommendation rather vague. Who should fund this? What does a new paradigm look like? How realistic is this?

>> I don't propose to have the solution to this problem. I'm not sure what that model would look like, but between philanthropists, crowd funding, non-profits, and federal agencies, I'd like to think some creative solutions could emerge.

>> I edited the draft to indicate that the solutions to this are currently unclear, and that it may take creative new solutions involving the parties I mentioned above. (new text red)

We may need new funding paradigms to support small open source teams. The form of these funding paradigms is not fully clear. Hackathons and similar forms of focused, small-team collaboration could possibly be sponsored through existing federal and philanthropic mechanisms for meetings and travel grants. Crowdsourcing could potentially fund some focused community tool development and maintenance. There is also room for creativity among funding organizations for smaller grants with faster review cycles for community tool building efforts.

3. For the the section "Shared tools to configure models and explore data", I wonder if new platforms and resources such as Binder and Code Ocean offer possible paths to progress. If code and data accompanying a given paper are in a runnable container available for all to easily tinker with and rerun, modifying parameters, doesn't that change much? Moreover, if I can swap easily one dataset with another, and hit "run" again in the cloud without having to install the entire pipeline, how much more replication and follow up does that encourage?

>> These are great points. I think this helps greatly for reproducibility, but doesn't necessarily help for creating a broader ecosystem of modular tools that can be connected into greater workflows, or augmented by new tools. Offering existing data and existing tools for rerunning is great but limited. We also seek to encourage "remixes" that are difficult to do in a single binder or virtualization. So, these are good solutions that should be mentioned, but they don't get at the ecosystem I think we can reach.

>> Moreover, they don't solve the problems of making (sophisticated) models user-friendly and easy to configure to *new* problems. (Sure, omics people have standard tools, but multicellular modeling people do not as much.)

I modified the "current progress" text to mention these great possibilities and their limitations (new text in red):

… not used to visualize biological data.

Cloud-hosted tools have provided a means to share sophisticated tools with broad, multidisciplinary audiences without need for downloading and compiling the tools. For example, the National Cancer Institute (NCI) has introduced NCI cloud resources as part of the NCI Cancer Research Data Commons [ref]. Sophisticated simulation models can also be shared as web applications: the PhysiCell development team recently created xml2jupyter

[ref]to automatically create Jupyter-based graphical user interfaces (GUIs) for PhysiCell-based multicellular simulations, which can then be cloud-hosted on platforms like nanoHUB [ref].

Other model and data sharing paradigms that emerged to address related issues in reproducibility may also encourage reuse, such as bundling data and software with Binder [ref] or *GigaScience*'s recent partnership with CodeOcean to pair papers with cloud-hosted executable platforms [ref]. However, these typically are single-purpose workflows (specialized to a specific data analysis for a single paper) that are not designed for modular reuse in new research workflows. They tend to lack standardized data formats (see Challenge 1 and Challenge 2) to facilitate connection with other tools, and latency issues will challenge their applicability in high-throughput workflows. While cloud-hosted executable codes increase accessibility and availability, they must not substitute for (or circumvent) sharing source code for full reproducibility.

>> In the "future" section, I added this text to further address these points:

Lastly, shared code platforms such as the NCI Data Commons could provide an environment to connect data and tools in online, easy-to-use workflows that encourage scientists to “mix and match” data software components into unique research. However, it will be important to avoid “lock-in” effects that prevent moving data and tools from one platform to another. Moreover, as workflows come to incorporate more web services (in differing platforms), they could become vulnerable to technical failures, business failures, or malicious attacks. Open source software has largely solved these issues by mirroring software repositories. Web services may need similar mirroring, and open science norms will need to encourage source code sharing and data/tool portability for web platforms just as they have for offline code.

4. In the section "Community-curated public data libraries", the author writes:
////////////
"An unfortunate consequence of the current data hosting model is that all the burden rests on data donors: they generate the data, format it to standards, assemble it, document it, upload it, and then pay the hosting and scientific publication costs. This is a classic case of the tragedy of the commons: it is easy to benefit from shared resources, but costly to contribute. Most repositories have fee waivers for scientists in low-income nations, but small and underfunded labs and citizen scientists are still at a disadvantage…

We need to develop more unified, financially stable and scalable repositories that can bridge fields and collect our knowledge. The repositories need to be community curated and continually improved, rather than static. They need to place less effort and financial burden on those who are donating data."
///////////

I personally find this part unconvincing. Of course, it would be fantastic if depositing in Dryad would be free, but is their charge of $120 per dataset really an unreasonable burden? The authors depositing there have often spent hundreds of thousands of dollars generating the datasets themselves. They are likely to pay thousands of dollars in page charges and article publishing charges. But the $120 fee for hosting and curating huge datasets is a "tragedy"?

>> First, please note that "tragedy" is not a word of my own invention; "tragedy of the commons" is a well-established term for shared-resource systems where individual agents act to personal benefit by draining a shared resource, and where it is easier to benefit than contribute. In the context of shared data and tools, donors bear the costs (development costs, helping competitors, data hosting costs, opportunity cost such as not having more time to write grants, etc.), whereas all others get the benefits without helping to support the donors.

>> This is the argument I'm making here. While bench scientists may get big grants and can easily pay these sharing costs (although they often don't – see big labs that publish in "top tier" journals, don't share data, and don't even pay the open access fee so others can easily read it), groups doing secondary analyses (or generating large *in silico* datasets) often don't have such large funds.

Paul Macklin, Ph.D. • **web**: MathCancer.org • **mobile**: 626-372-1203 • **Twitter**: @MathCancer • macklinp@iu.edu
Dept. of Intelligent Systems Engineering • Indiana University • Luddy Hall 4128, 700 N. Woodlawn Ave, Bloomington, IN 47408

Ideally, those who run a simulation model that generates 10-100 GB of data over a few weeks of computing would like to share that dataset, but they may well not have extra funds to do that. (I've been in this situation myself.) In this case, the reproducibility-minded lab has (1) paid for the research out-of-pocket (or startup), (2) paid the open access publication fees, and then (3) pays the data hosting fees on top. For a small computational lab with a $100,000-$250,000 (this a typical startup budget for an tenure-track assistant professor performing simulations), these are significant extra costs to "do the right thing," and while the costs are dropping, they should not be considered lightly.

>> I have better emphasized that "tragedy of the commons" is a phrase (and not a literal tragedy) by placing it italics and quotes in the text, while also softening the tone (changes in red):

> This is a classic case of the *"tragedy of the commons"*: it is easy to benefit from shared resources, but the cost of contribution falls on contributors. ~~costly to contribute.~~ Most repositories have fee waivers for scientists in low-income nations, but small and underfunded labs and citizen scientists are still at a disadvantage.
>
> Nonprofit organizations like DRYAD have made great strides in creating sustainable resources to host data; currently (as of 2019), a one-time charge of US $120 per dataset applies once the data are accepted by curators and publicly available [ref]. This is a small fee compared to the data generation cost for experimental labs and within the means of well-funded labs.

Comparison to bioRxiv is not appropriate as bioRxiv is dealing with minuscule PDF files rather than gigabytes of data. Moreover, bioRxiv does not curate in contrast to Dryad. Finally, in calling for more "financially stable and scalable repositories", how would not charging and relying on even more on philanthropic grant support make a repository like Dryad more stable and sustainable?

>> I agree that DRYAD have made good advances towards sustainable data hosting. I revised to account for these great points as follows (changes in red):

> We need to develop more unified, ~~financially stable and~~ scalable repositories that can bridge fields and collect our knowledge. The repositories should ~~need to~~ be indexed and, community curated ~~, and continually improved, rather than static~~ to encourage continuous refinement where possible. While there has been great progress to create financially sustainable, permanent data hosting, there is still room to explore alternative funding for data generated independently of specific grant funds. Moreover, these archive-oriented data stores still require curation and indexing if they are to grow from data storage to libraries.
>
> Solutions to this challenge may well originate outside the bioinformatics community. Library scientists have longstanding domain expertise in collecting and curating knowledge across disciplines in unified physical libraries: this expertise would undoubtedly benefit any efforts to create public data libraries. The tremendous success of Wikipedia [41] in hosting its own image and video resources on Wikimedia Commons [42]—at no cost to contributors—could be a very good model. bioRxiv [43] has been similarly successful in hosting preprints at no cost to authors, although experimental data hosting costs are far higher than the cost of hosting manuscripts. Both of these have relied upon a combination of public donations, federal support, and philanthropy, channeled through appropriate nonprofit structures.

In addition, the call, "The repositories need to be community curated and continually improved, rather than static", also seems like wishful thinking to me. I understand that versioning and support for sharing improvements is a must. At the same time, I caution against an expectation of "continually improved rather than static" on a large scale. These datasets take enormous resources and often are static because funding runs out or the people producing them move on. Of course, resources such as genome assemblies from mega-consortiums have improvements and new releases, but for most individual labs, the burden of "continuous improvement" is simply not realistic.

Paul Macklin, Ph.D. • **web**: MathCancer.org • **mobile**: 626-372-1203 • **Twitter**: @MathCancer • macklinp@iu.edu
Dept. of Intelligent Systems Engineering • Indiana University • Luddy Hall 4128, 700 N. Woodlawn Ave, Bloomington, IN 47408

>> While I agree that primary data will largely remain static (and expecting refinement of published primary results is often non-practical), secondary analyses and overall indexing and curation will be benefit from continuous community refinement. I revised as:

> Public data libraries should enable if not encourage versioned post-publication refinement, particularly for datasets arising from secondary analysis or curation of heterogeneously sourced primary raw data, such as digital cell lines [ref].

5. What are other challenges, beyond the eight described here? For example, one serious issue is lack of standard methods for creating datasets. Different labs may use distinct approaches and techniques in preparing and analyzing samples. Even when the technique is largely the same, the devil is often in the details - slight deviations in specific steps (for example "30 degrees" versus "room temperature") may lead to significant differences in results/data. These deviations can then be create a misleading appearance of real phenotypic differences between tissues studied such as spleen versus liver, or healthy versus oncogenic samples.

This is just one issue that does not seem to be covered in the current review, and I am sure there are others. Perhaps mentioning these other factors in the conclusion/discussion section would be useful?

>> I agree with the reviewer that there may well be other challenges. The manuscript explicitly encourages readers to identify other challenges.

Preprint Comments 1: (Assaf Zaritsky)

Just finished enjoying reading your review. This is the first time I hear the term multicellular systems biology, I hope that it will catch up :-) I especially like the ideas of mining through high-content simulated data and agree that this has vast potential to teach us new biology.

>> Thank you!

As it is in its preprint stage, I allow myself to make a few suggestions, mostly related to related previous work you might consider mentioning:

Would be nice to hear about achievements of multicellular systems biology, I know the field is nascent, but some work have been done, both from theoretical simulations and from the data-modeling perspective. Making the point that the field is emerging has to come with examples. I can think of a few examples (including my own work in the context in the context of data reuse and big data analysis ;-)) I can provide with some references, if you'd like.

>> Thank you! I'd like to get into reviews of the merits and successes of multicellular systems biology, but I think it's a bit out of scope for this paper. (But it's a nice idea!) But I think it could be a good article. Maybe this is something we could jointly interest the staff at GS in??

In the context of repositories I would highly recommend mentioning Jason Swedlow's IDR (https://idr.openmicroscopy.org/about/, https://www.nature.com/articles/nmeth.4326).

>> Really great point! I added the reference. (new text in red):

> … Cancer Genome Atlas [ref].
>
> The Image Data Resource [ref] was recently launched to facilitate sharing bioimages using the OME data format [ref], further demonstrating how standardized data can facilitate the creation of shared tools and resources.

In the context of data quality and curation standards I point you to the white paper by Jason and Jan Ellenberg https://arxiv.org/abs/1801.10189

>> Thank you. I found the published version in Nature Methods and cited it in Challenge 6.

Open data ecosystem - you can mention MULTIMOT (EU funded), https://multimot.org, and the community efforts in standardization in https://multimot.org/. Both in the context of cell migration. CMSO are currently in the process of writing a manuscript and I just sent your preprint to the lead author, as I think it should be mentioned there..

>> Thanks for reminding me about the excellent MULTIMOT work. I added this to the tail end of the "current progress" part of Challenge 1:

> The European Union-funded MULTIMOT project [ref] has been developing a community-driven standard for cell motility measurement (MIACME: Minimum Information about Cell Migration Experiments), with a corresponding software ecosystem that can interface with data in ISA-Tab and OME formats.

And also here:

> Ultimately, we should combine and extend them into a unified data format. ISA-Tab could bundle image data (using OME) and extracted biological features (e.g., with MultiCellDS and MULTIMOT), while storing experimental protocol details with a controlled vocabulary growing out of Protocols.IO [ref].

Also, my recent perspective also has many parallels with the ideas and challenges you present, https://www.molbiol-cell.org/doi/abs/10.1091/mbc.E17-10-0606, would be nice to be mentioned, if you find it appropriate, of course.

>> Thank you, this is a great article! I cited it in Challenges 4 and 6.

Cheers, enjoy the rest of the weekend,

>> Thank you for your helpful suggestions!

Preprint Comments 2: (Alejandra Gonzalez-Beltran)

Dear Paul,

I am writing to you w.r.t. your review article on "Key challenges facing data-driven multicellular systems biology". I spoke with Scott about how your article is related to the work we've been doing within the EU MultiMot project (https://multimot.org/) and have been meaning to write to you since then.

>> Thank you for writing!

The MultiMot project created the Cell Migration Standardisation Organisation (CMSO, http://cmso.science/), in which we have been developing standards for reporting cell migration experiments. These standards include MIACME (Minimum Information about Cell Migration Experiments) as a reporting guideline that can be integrated with ISA-Tab (I am part of the ISA team), the use of OME and a new format for cell tracking data called biotracks.

>> Thank you – I'm very excited about your work and project! I cited this excellent work in Challenge 1:

> The European Union-funded MULTIMOT project [ref] has been developing a community-driven standard for cell motility measurement (MIACME: Minimum Information about Cell Migration Experiments), with a corresponding software ecosystem that can interface with data in ISA-Tab and OME formats.

So, I wanted to reach out to make you aware of this work, as we hope addresses some of the challenges in terms of standards that you pointed out in your article. We are about to make available a pre-print of the work done by CMSO and will let you know when it is out.

>> Fantastic! I hope that you can one day publish some of that work here!

It would be good if you let me know about the status of your pre-print, as we are referring to it. If there is time, it would be great if you could highlight the work we've been doing at the CMSO.

>> Absolutely! Thank you!
