# Supplementary material for: Key challenges facing data-driven multicellular systems biology
Source: Gigascience. 2019 Oct 24;8(10):giz127. doi: 10.1093/gigascience/giz127 (PMC6812467; doi:10.1093/gigascience/giz127)
Supplement: giz127_Response_to_Reviewer_Comments_Original_Submission [file giz127_response_to_reviewer_comments_original_submission.pdf]

Author's Response To Reviewer Comments

Close

Please see attached letter.

Close
