# Supplementary material for: Key challenges facing data-driven multicellular systems biology
Source: Gigascience. 2019 Oct 24;8(10):giz127. doi: 10.1093/gigascience/giz127 (PMC6812467; doi:10.1093/gigascience/giz127)
Supplement: giz127_Reviewer_1_Report_Original_Submission — Brett Beaulieu-Jones -- 7/1/2018 Reviewed [file giz127_reviewer_1_report_original_submission.pdf]

Reviewer Report

Title: Key challenges facing data-driven multicellular systems biology

Version: Original Submission **Date:** 7/1/2018

Reviewer name: Brett Beaulieu-Jones

Reviewer Comments to Author:

This review discusses core issues data-driven multicellular biology faces, given rapid advances in the field (high-throughput measurement/manipulation technologies and more complex modelling). It raises questions around reproducibility, data sharing and interoperability to encourage collaboration as well as how to structure what is learned. These are unsolved problems in many fields and especially important in data-driven multicellular biology given it's complexity of experiments and data.
Within the Key Challenges, it states: "We need to move beyond shared drives of raw images and spreadsheets to extracted biological elements that are useful for building models and machine learning."
I would argue that this is insufficient without also including the raw data and detailed metadata explaining how biological elements are extracted. Tools and technologies (and annotations) change rapidly over time which can lead to significant differences in extracted data.
In addition to protocols.io I would also mention some of the tools that allow you to export a computational pipeline to ensure others could reproduce your (via docker, jupyter notebooks etc.).
Under shared multicellular representations: An interesting parallel would be some of the tools to extract genetic associations and/or relationships from literature. These are commonly stored in graph databases.
Under community-curated public data libraries: A final point is the idea that if we create many commons, nothing is common between them. By that I mean, it becomes hard to know where to go for what. I think it'd be helpful to further emphasize the need standards and community agreement around which data libraries serve as standards.
Overall, this is a nice review of some of the challenges facing a fast-moving field that is of growing importance.

Methods

Are the methods appropriate to the aims of the study, are they well described, and are necessary controls included? Choose an item.

Conclusions

Are the conclusions adequately supported by the data shown? Choose an item.

Reporting Standards

Does the manuscript adhere to the journal's guidelines on [minimum standards of reporting?]() Choose an item.

Choose an item.

Statistics

Are you able to assess all statistics in the manuscript, including the appropriateness of statistical tests used? Choose an item.

Quality of Written English

Please indicate the quality of language in the manuscript: Choose an item.

Declaration of Competing Interests

Please complete a declaration of competing interests, considering the following questions:

- Have you in the past five years received reimbursements, fees, funding, or salary from an organisation that may in any way gain or lose financially from the publication of this manuscript, either now or in the future?
- Do you hold any stocks or shares in an organisation that may in any way gain or lose financially from the publication of this manuscript, either now or in the future?
- Do you hold or are you currently applying for any patents relating to the content of the manuscript?
- Have you received reimbursements, fees, funding, or salary from an organization that holds or has applied for patents relating to the content of the manuscript?
- Do you have any other financial competing interests?
- Do you have any non-financial competing interests in relation to this paper?

If you can answer no to all of the above, write 'I declare that I have no competing interests' below. If your reply is yes to any, please give details below.

I declare that I have no competing interests.

I agree to the open peer review policy of the journal. I understand that my name will be included on my report to the authors and, if the manuscript is accepted for publication, my named report including any attachments I upload will be posted on the website along with the authors' responses. I agree for my report to be made available under an Open Access Creative Commons CC-BY license (http://creativecommons.org/licenses/by/4.0/). I understand that any comments which I do not wish to be included in my named report can be included as confidential comments to the editors, which will not be published.

Choose an item.

To further support our reviewers, we have joined with Publons, where you can gain additional credit to further highlight your hard work (see: https://publons.com/journal/530/gigascience). On publication of this paper, your review will be automatically added to Publons, you can then choose whether or not to claim your Publons credit. I understand this statement.

Yes Choose an item.
