# Supplementary material for: Key challenges facing data-driven multicellular systems biology
Source: Gigascience. 2019 Oct 24;8(10):giz127. doi: 10.1093/gigascience/giz127 (PMC6812467; doi:10.1093/gigascience/giz127)
Supplement: giz127_Reviewer_2_Report_Original_Submission — Lenny Teytelman -- 7/2/2018 Reviewed [file giz127_reviewer_2_report_original_submission.pdf]

Reviewer Report

Title: Key challenges facing data-driven multicellular systems biology

Version: Original Submission **Date:** 7/2/2018

Reviewer name: Lenny Teytelman

Reviewer Comments to Author:

This is a review of "Key challenges facing data-driven multicellular systems biology".
My most important note is that I am a very imperfect reviewer for it. I am no expert in systems biology and as a yeast geneticist, I am certainly no guru in multicellular systems and challenges. Therefore, while I very much enjoyed the paper, I hope that others have reviewed it who are more familiar with this research topic.
Below are some suggestions that the auhor might want to consider.
1. The paper describes 8 "Key challenges" facing the field. Are all of these equal barriers to progress or are some more serious than others? Ranking these and highlighting which of them are indeed critical to address would be useful. As is, a reader might conclude that true progress in the field is hopeless. Moreover, if the paper is meant to serve as a call to action for funders and the community, it would be beneficial to opine in the discussion section which challenges deserve the most funding/attention. If we had to pick one of these eight to tackle, would working towards standards be most important or is focus on developing good benchmarking datasets a better first target?
2. The section "Shared tools to configure models and explore data" ends with, "We may need new funding paradigms to support small open source teams." I find this recommendation rather vague. Who should fund this? What does a new paradigm look like? How realistic is this?
3. For the the section "Shared tools to configure models and explore data", I wonder if new platforms and resources such as Binder and Code Ocean offer possible paths to progress. If code and data accompanying a given paper are in a runnable container available for all to easily tinker with and rerun, modifying parameters, doesn't that change much? Moreover, if I can swap easily one dataset with another, and hit "run" again in the cloud without having to install the entire pipeline, how much more replication and follow up does that encourage?
4. In the section "Community-curated public data libraries", the author writes:
////////////
"An unfortunate consequence of the current data hosting model is that all the burden rests on data donors: they generate the data, format it to standards, assemble it, document it, upload it, and then pay the hosting and scientific publication costs. This is a classic case of the tragedy of the commons: it is easy to benefit from shared resources, but costly to contribute. Most repositories have fee waivers for scientists in low-income nations, but small and underfunded labs and citizen scientists are still at a disadvantage…
We need to develop more unified, financially stable and scalable repositories that can bridge fields and collect our knowledge. The repositories need to be community curated and continually improved, rather than static. They need to place less effort and financial burden on those who are donating data."

///////////
I personally find this part unconvincing. Of course, it would be fantastic if depositing in Dryad would be free, but is their charge of $120 per dataset really an unreasonable burden? The authors depositing there have often spent hundreds of thousands of dollars generating the datasets themselves. They are likely to pay thousands of dollars in page charges and article publishing charges. But the $120 fee for hosting and curating huge datasets is a "tragedy"?
Comparison to bioRxiv is not appropriate as bioRxiv is dealing with minuscule PDF files rather than gigabytes of data. Moreover, bioRxiv does not curate in contrast to Dryad. Finally, in calling for more "financially stable and scalable repositories", how would not charging and relying on even more on philanthropic grant support make a repository like Dryad more stable and sustainable?
In addition, the call, "The repositories need to be community curated and continually improved, rather than static", also seems like wishful thinking to me. I understand that versioning and support for sharing improvements is a must. At the same time, I caution against an expectation of "continually improved rather than static" on a large scale. These datasets take enormous resources and often are static because funding runs out or the people producing them move on. Of course, resources such as genome assemblies from mega-consortiums have improvements and new releases, but for most individual labs, the burden of "continuous improvement" is simply not realistic.
5. What are other challenges, beyond the eight described here? For example, one serious issue is lack of standard methods for creating datasets. Different labs may use distinct approaches and techniques in preparing and analyzing samples. Even when the technique is largely the same, the devil is often in the details - slight deviations in specific steps (for example "30 degrees" versus "room temperature") may lead to significant differences in results/data. These deviations can then be create a misleading appearance of real phenotypic differences between tissues studied such as spleen versus liver, or healthy versus oncogenic samples.
This is just one issue that does not seem to be covered in the current review, and I am sure there are others. Perhaps mentioning these other factors in the conclusion/discussion section would be useful?

Methods

Are the methods appropriate to the aims of the study, are they well described, and are necessary controls included? Choose an item.

Conclusions

Are the conclusions adequately supported by the data shown? Choose an item.

Reporting Standards

Does the manuscript adhere to the journal’s guidelines on minimum standards of reporting? Choose an item.

Choose an item.

Statistics

Are you able to assess all statistics in the manuscript, including the appropriateness of statistical tests used? Choose an item.

Quality of Written English

Please indicate the quality of language in the manuscript: Choose an item.

Declaration of Competing Interests

Please complete a declaration of competing interests, considering the following questions:

- Have you in the past five years received reimbursements, fees, funding, or salary from an organisation that may in any way gain or lose financially from the publication of this manuscript, either now or in the future?
- Do you hold any stocks or shares in an organisation that may in any way gain or lose financially from the publication of this manuscript, either now or in the future?
- Do you hold or are you currently applying for any patents relating to the content of the manuscript?
- Have you received reimbursements, fees, funding, or salary from an organization that holds or has applied for patents relating to the content of the manuscript?
- Do you have any other financial competing interests?
- Do you have any non-financial competing interests in relation to this paper?

If you can answer no to all of the above, write 'I declare that I have no competing interests' below. If your reply is yes to any, please give details below.

I am one of the co-founders of protocols.io and hold equity in the company. protocols.io is mentioned in this manuscript.

I agree to the open peer review policy of the journal. I understand that my name will be included on my report to the authors and, if the manuscript is accepted for publication, my named report including any attachments I upload will be posted on the website along with the authors' responses. I agree for my report to be made available under an Open Access Creative Commons CC-BY license (http://creativecommons.org/licenses/by/4.0/). I understand that any comments which I do not wish to be included in my named report can be included as confidential comments to the editors, which will not be published.

Choose an item.

To further support our reviewers, we have joined with Publons, where you can gain additional credit to further highlight your hard work (see: https://publons.com/journal/530/gigascience). On publication of this paper, your review will be automatically added to Publons, you can then choose whether or not to claim your Publons credit. I understand this statement.

Yes Choose an item.
